# DNA-based assessment of root lesion nematode infections in cereal roots

**DOI:** 10.1038/s41598-023-39559-8

**Published:** 2023-08-03

**Authors:** Ehsan Fatemi, Siegbert Melzer, Christian Jung

**Affiliations:** Plant Breeding Institute, Christian-Albrechts-University of Kiel, Olshausenstr. 40, 24098 Kiel, Germany

**Keywords:** Ecology, Microbiology, Molecular biology, Plant sciences, Environmental sciences

## Abstract

Root lesion nematodes (RLN) of the genus *Pratylenchus* are causing significant damage in cereal production worldwide. Due to climate change and without efficient and environment-friendly treatments, the damages through RLNs are predicted to increase. Microscopic assessments of RLNs in the field and the greenhouses are time-consuming and laborious. As a result, cereal breeders have mostly ignored this pest. We present a method measuring RLN in infected cereal roots using a standardized PCR approach. Publicly available *Pratylenchus neglectus* primer combinations were evaluated. An optimal primer combination for RT-qPCR assay was identified to detect and quantify *P. neglectus* within infected cereal roots. Using the RT-qPCR detection assay, *P. neglectus* could be clearly distinguished from other plant parasitic nematodes. We could identify *P. neglectus* DNA in barley and wheat roots as low as 0.863 and 0.916 ng/µl of total DNA, respectively. A single *P. neglectus* individual was detected in water suspension and within barley and wheat roots. The RT-qPCR detection assay provides a robust and accurate alternative to microscopic nematode identification and quantification. It could be of interest for resistance breeding, where large populations must be screened to detect and quantify *P. neglectus* in farmer’s fields.

## Introduction

Approximately 4100 plant parasitic nematode (PPN) species are known^1^. Many are devastating pests in agriculture and horticulture worldwide^2,3^. For example, the worldwide yield loss caused by crop PPNs has been projected to be approximately 15%, with losses in some regions exceeding 50%^4–6^.

Root-lesion nematodes (RLN) of the genus *Pratylenchus* are the third most damaging PPNs to crops worldwide, after root-knot and cyst nematodes^7,8^. The damage from RLN is affected by various factors, including nematode species involved, climate, host range, nematode virulence, and cropping systems. Studies have reported yield losses caused by RLNs ranging from 5 to 50% in various crops depending on the crop and the severity of the infestation^2,7^.

*Pratylenchus neglectus* and *P. thornei* are the two most important RLNs in cereals. The *P. neglectus* population in the soil negatively correlates with wheat grain yield^9^. In Australia, wheat yield can be reduced by up to 30%^10^. *P. neglectus* was detected in more than 90% of dryland wheat fields in the United States Pacific Northwest, where their damage is estimated to be $51 million per year^11–13^. In Europe, farmers are using narrow crop rotations and early sowing dates, which cause increasing damage by RLNs^14,15^. A survey of PPNs in organic farming in Germany discovered the genera *Pratylenchus* and *Tylenchorhynchus* in over 90% of collected soil samples^16^. RLNs have often been overlooked because of their species diversity, migratory behavior, morphological similarities, similar damage symptoms by other soilborne pathogens or environmental stresses, and lack of trained nematologists^17^. Identifying nematodes based on morphological traits is time-consuming and requires immense expertise for nematode classification. The quantification process takes time, and counting and identifying these species from many samples is challenging, especially when other nematodes are present. For instance, distinguishing *P. neglectus* from *P. thornei* and other closely related *Pratylenchus* spp. is based on minor morphological characteristics in lip annule number, tail shape, and vulva position^18^. Furthermore, species identification can be complicated by environmental conditions and phenotypic variation^13,19^. Due to these challenges, there is a need to develop simple and quick diagnostic strategies for identifying nematodes. Combining morphological and molecular data will be ideal for improving the resolution and reliability of diagnostic studies^20^.

DNA-based nematode diagnostics have been established as a fast alternative to microscopic analyses. Commercial laboratories offer comprehensive DNA-based testing for quantifying various soilborne diseases^21,22^. However, the details of the used protocols are considered proprietary and not openly available. However, PCR-based approaches for differentiating PPN species have been developed. For example, a method using the cytochrome c-oxidase subunit I gene (*mtCOI*) and the Internal Transcribed Spacer sequences (ITS) could identify potato cyst nematodes and track their distribution in Indonesia^23^. Likewise, *mtCOI* primer combinations have distinguished four *Aphelenchoides* species^24^. Another study demonstrated using *mtCOI* and 18S rRNA-specific primer combinations to identify marine nematodes and provide voucher specimens. This preserved specimen serves as a verifiable and permanent record of nematodes^25^. Boroş et al. used primer combinations binding to the cytochrome oxidase II (*mtCO2*) and the 16S rRNA genes to identify four different *Meloidogyne* species^26^. Most PCR-based molecular diagnostic techniques identify *Pratylenchus* spp. by utilizing species-specific ribosomal DNA (rDNA) polymorphisms. Molecular diagnostic tests for a wide range of *Pratylenchus* spp. have been established, primarily based on tandemly arranged rDNA genes present in many copies in the genome^27,28^. Thereby, RT-qPCR approaches for identifying *P. vulnus* from pure culture and soil^29,30^ and randomly amplified polymorphic DNA (RAPD) fragments to identify *P. thornei* from nematode isolates on carrot discs^31^ have been developed. An RT-qPCR test based on SYBR^®^ Green-I to detect and quantify the root lesion nematode *P. zeae*, the root-knot nematode *Meloidogyne javanica*, and the dagger nematode *Xiphinema elongatum* from soil^32^ were established. Moreover, RT-qPCR-based protocols were developed for detecting *P. penetrans*^33^ and *P. thornei*^34^ from soil DNA samples.

Al-Banna, et al.^27^ reported differentiating six *Pratylenchus* spp., including *P. neglectus* and *P. thornei*, from soil samples by PCR. They designed a species-specific primer combination within the D3 expansion domain of 26S rDNA in a conventional PCR-based assay to detect *P. neglectus*. Likewise, Yan, et al.^28^ designed primer combinations from the same genome region to identify *P. neglectus* and *P. thornei* from soil samples, albeit with low sensitivity. Therefore, Yan, et al.^35^ developed a new primer combination for detecting and quantifying *P. neglectus* in soil within the ITS1 and 5.8S regions using a SYBR^®^ Green-I-based RT-qPCR method. Using publicly available sequence information, Peetz and Zasada^36^ designed species-specific primer combinations based on the *β-1,4-endoglucanase* gene for different *Pratylenchus* species (*P. crenatus, P. neglectus, P. penetrans,* and *P. thornei*) which they applied for soil monitoring in the Pacific Northwest of North America. Three more studies used a TaqMan-based RT-qPCR with species-specific primer combinations for detecting *P. neglectus*. Jayatilake, et al.^37^ designed a species-specific primer combination from 28S large subunit ribosomal rDNA to detect and quantify *P. neglectus* within infected wheat roots. Oliveira, et al.^38^ reported an RT-qPCR assay-based assay for detecting *P. crenatus*, *P. penetrans*, and *P. neglectus* in soil samples. They calculated the number of ITS-1 copy numbers per *P. neglectus* nematode. Finally, Lin, et al.^39^ established a duplex real-time qPCR with a species-specific primer combination within the D2D3 expansion domain of 28S rDNA for detecting and quantifying *P. neglectus* and *P. thornei* in soil samples which was sensitive enough for detecting a single nematode among a population of non-target nematodes.

All published methods were designed for *P. neglectus* detection and quantification in water suspension and soil samples. Moreover, DNA isolation and RT-qPCR-based protocols for detecting and quantifying *P. neglectus* within cereal roots are lacking. Therefore, we performed a comparative study using published primer combinations to establish a protocol for quick and routine detection and quantification of *P. neglectus* within infected cereal roots. Moreover, we established a protocol for DNA extraction from infected roots and performed a series of experiments to select the most effective primer combination for *P. neglectus* detection. This low-cost method allows a precise, sensitive, and efficient diagnosis and identification of *P. neglectus* within plant root tissues.

## Results

### Evaluating primer combinations suitable for RT-qPCR

We searched the literature and the Genbank database for *P. neglectus*-specific primer combinations (Supplementary Table 2). The sequence flanking the Neg1 primer^35^ was identical to all available *P. neglectus* sequences, including accession KY468880.1 which had been submitted as *P. crenatus*. The morphological description of this isolate has not been published, but its sequence is distinct from all other *P. crenatus* Genbank sequences but highly similar to *P. neglectus*. The sequence flanking the Neg2 primer^36^ showed only a few *P. neglectus* BLAST hits and was therefore excluded from further analysis. The sequence from the 28S rRNA D3 expansion flanking the Neg3 primer^28^ was also discarded because it shares high similarity to sequences of other *Pratylenchus* species such as *P. minyus*, *P. kumamotoensis*, and *P. vulnus*. The Neg4 forward primer sequence^27^ (Neg4-fw; Supplementary Table 2) did not show any BLAST hits because it lacked a primer nucleotide at position 13. However, inserting an adenine at this position resulted in a primer sequence with 100% identity to all *P. neglectus* sequences from the database.

### PCR and RT-qPCR experiments with different plant parasitic nematode species

**Figure 1:**
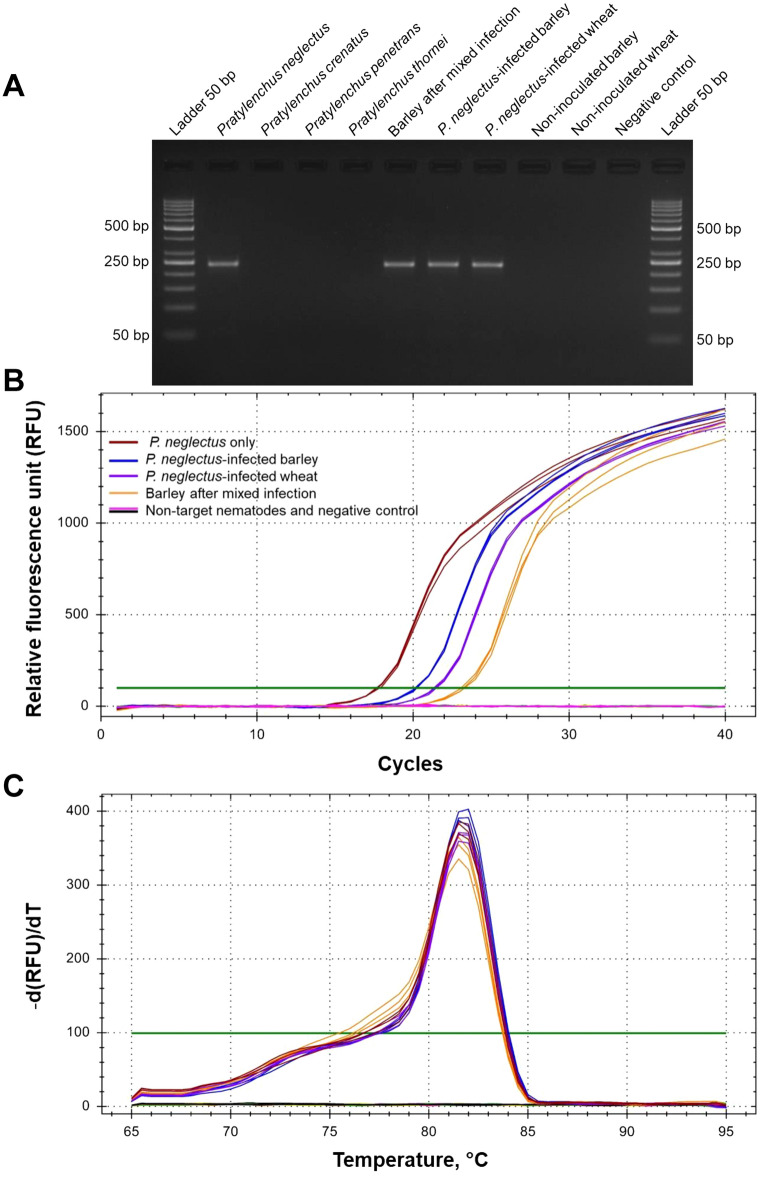
Results of the PCR and RT-qPCR experiments with RLN DNA using the Neg1 primer combination. The primer sequences, annealing temperatures, and the expected fragment sizes are given in Supplementary Table 2, and the material and method. (**A**) Agarose gel (3%, 80 V for 60 min) with PCR fragments amplified with DNA from different RLN species and cereal roots infected with *P. neglectus*, (**B**) RT-qPCR amplification curves with DNA from *Pratylenchus neglectus*, cereal roots infected with *P. neglectus*, other *Pratylenchus* species, and non-infected roots. Each data point represents the mean of three technical replicates. (**C**) Melting curve profiles of *P. neglectus*-specific amplicons. The peak is at 81.5 °C. None of the non-*P. neglectus* amplification curves touch the threshold line.

All primer combinations were tested with four populations of different species of *P. neglectus* DNA separately. Total DNA from barley and wheat inoculated with either *P. neglectus* alone or a mixture of all four species were used as controls (Supplementary Table 1; Supplementary Table 3). As a result, the Neg1 primer combination gave the expected PCR product of 234 bp (Fig. 1A), while no amplification product was found with non-target nematode DNA. It showed the most reliable PCR results because of strong and distinct bands in the presence of *P. neglectus* DNA (Fig. 1A). Amplification curves (Fig. 1B) and a single melting peak at 81.5 °C (Fig. 1C) for samples containing pure *P. neglectus* DNA, DNA from *P. neglectus* inoculated barley and wheat, and DNA from barley inoculated with a mixture of different RLN species, demonstrate the potential of this primer combination for amplifying *P. neglectus* DNA from different sources.

A *P. neglectus*-specific PCR product of 293 bp was generated with the Neg2 primer combination, with visible primer dimers in the absence of a DNA template (Supplementary Fig. 1). In addition, the amplification curves revealed a lower detection sensitivity, with two to three cycles more before reaching the threshold (Supplementary Fig. 2A; Supplementary Table 3). The melting curve analyses showed a single peak at 88.5 °C (Supplementary Fig. 2B). The Neg3 primer combination gave a PCR product of 144 bp, but primer dimers were visible in the samples without template DNA (Supplementary Fig. 1). The RT-qPCR analyses revealed amplification curves and a lack of sensitivity in inoculated barley and wheat samples, with one to two cycles more before reaching the threshold (Supplementary Fig. 2C; Supplementary Table 3) with a single melting peak at 90.5 °C (Supplementary Fig. 2D). PCR with the Neg4 primer combination resulted in two amplicons, one band of the expected size of 290 bp and a second faint band of 200 bp (Supplementary Fig. 1). In addition, the amplification curves are roughly parallel, the curves are close to each other (Supplementary Fig. 2E; Supplementary Table 3), and the melting curve analysis revealed two peaks at 85 °C and 90.5 °C, indicating the amplification of a non-specific fragment (Supplementary Fig. 2F).

All PCR products were Sanger sequenced in both directions. All sequences showed high similarity to the expected PCR product sequences and a reference database of known sequences (data not shown).

### Quantification of nematode infections in cereal roots

First, we made serial dilutions of *P. neglectus* DNA (from non-diluted DNA, 1:0 to 1:1000) to check the sensitivity of the RT-qPCR assays using the DNA of 2000 *P. neglectus* individuals using the Neg1 primer combination (Table 1). The results showed a correlation between the DNA concentration of serial dilutions and the Cq values. The lowest, 16.55 ± 0.01, and highest, 28.87 ± 0.20, Cq values were obtained with 53.4 ng DNA/µl and 0.053 ng DNA/µl, respectively (Supplementary Fig. 5; Table 1). We also screened all PPN species with the Neg1 primer combination and effectively detected all *P. neglectus* isolates without detecting non-target nematodes (Supplementary Fig. 3, Supplementary Table 1).

**Table 1:** Quantification cycle (Cq) values from serial dilution experiments with pure *Pratylenchus neglectus* DNA (from 2000 nematodes) and DNA from barley and wheat plants inoculated with 1000 *P. neglectus*.

Serial dilutions	Samples	DNA (ng/µl)	Cq ± SD
Non-diluted DNA (1:0)	*P. neglectus*	53.4	16.55 ± 0.01
	Barley (infected)	863.4	20.93 ± 1.14
	Wheat (infected)	916.4	20.99 ± 1.04
1:2	*P. neglectus*	26.7	17.63 ± 0.04
	Barley (infected)	431.7	22.54 ± 0.24
	Wheat (infected)	458.2	22.64 ± 0.34
1:5	*P. neglectus*	10.68	18.96 ± 0.07
	Barley (infected)	172.68	24.08 ± 0.18
	Wheat (infected)	183.28	23.63 ± 0.02
1:10	*P. neglectus*	5.34	20.79 ± 0.19
	Barley (infected)	86.34	25.60 ± 0.25
	Wheat (infected)	91.64	24.54 ± 0.11
1:50	*P. neglectus*	1.07	22.04 ± 0.18
	Barley (infected)	17.27	26.68 ± 0.44
	Wheat (infected)	18.33	25.44 ± 0.27
1:100	*P. neglectus*	0.534	23.37 ± 0.05
	Barley (infected)	8.634	27.49 ± 0.13
	Wheat (infected)	9.164	26.61 ± 0.28
1:500	*P. neglectus*	0.107	26.24 ± 0.17
	Barley (infected)	1.726	28.21 ± 0.37
	Wheat (infected)	1.833	27.95 ± 0.24
1:1000	*P. neglectus*	0.053	28.87 ± 0.20
	Barley (infected)	0.863	29.02 ± 0.17
	Wheat (infected)	0.916	29.84 ± 0.54

The Neg1 primer combination was used for amplification.

*SD* standard deviation, calculated from three technical replicates.

**Figure 2:**
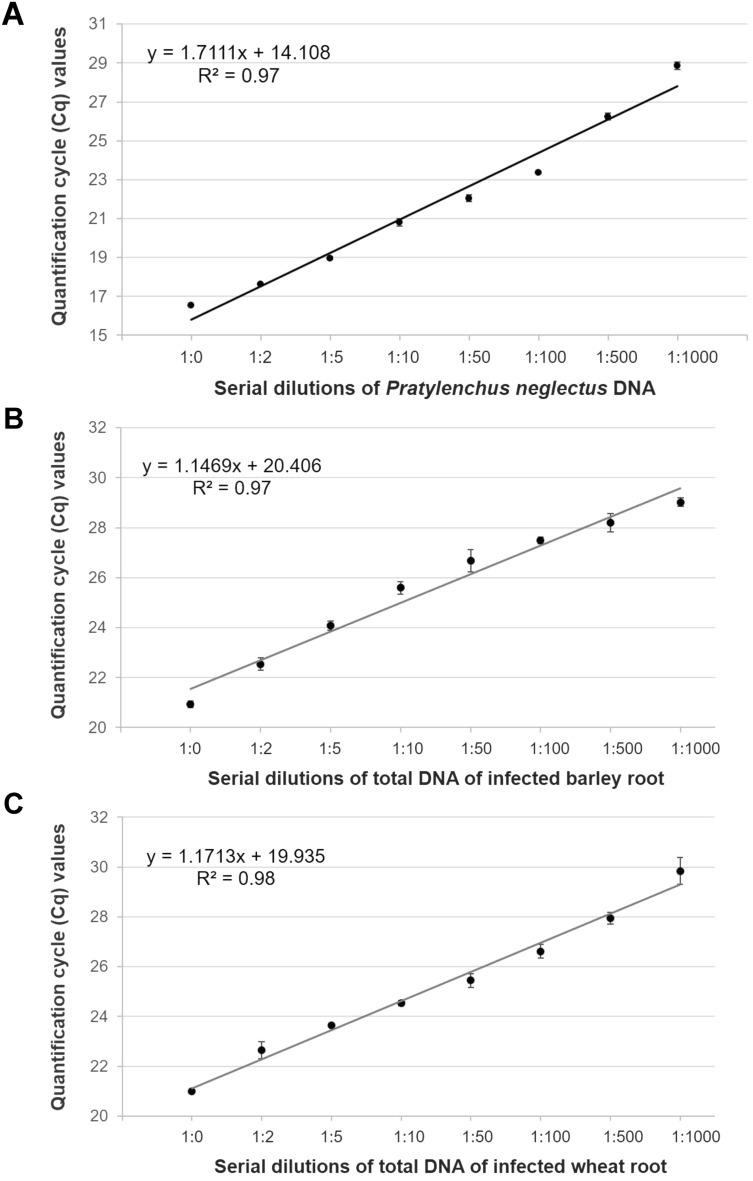
Quantification cycle (Cq) values plotted against serial dilutions of DNA. RT-qPCR was performed using the Neg1 primer combination. (**A**) Regression curve with serial dilutions of pure *P. neglectus* DNA extracted from 2000 individuals, (**B**) regression curve with serial dilutions of total DNA isolated from infected barley roots, (**C**) regression curve with serial dilutions of total DNA isolated from infected wheat roots. Each data point represents the mean of three technical replicates. Standard deviation indicates the variation between three technical replicates (see Table 1).

Then, barley and wheat plants were infected with 1000 *P. neglectus*. Eight weeks after inoculation, plants were harvested, and as described in the material and method, a modified DNA isolation method was used to isolate total DNA from infected roots. The obtained total DNA was diluted to 836.4 and 916.4 ng/µl for barley and wheat, respectively, for DNA quality check and PCR/RT-qPCR experiments (Table 1). PCR with Neg1 primer combination resulted in visible amplicons of the expected size (Supplementary Fig. 4). Then, we performed RT-qPCR with the same primer combination and a series of DNA dilutions (1:0–1:1000). The Cq values ranged from 20.93 ± 1.14 to 29.02 ± 0.17 for barley and 20.99 ± 1.04 to 29.84 ± 0.54 for wheat (Supplementary Fig. 5; Table 1). The efficiency of RT-qPCR for these serial dilutions was 87% for barley and 84% for wheat. The efficiency value indicates how much the RT-qPCR reaction can amplify the target nucleic acid sequence. The linear regression curves of DNA serial dilutions of *P. neglectus* and infected barley and wheat roots demonstrate that nematode DNA could be detected even within highly diluted root DNA (Fig. 2). High R^2^ values in all curves show a positive correlation between the DNA concentration from the serial dilutions and the Cq values. These results show that the RT-qPCR assay is sensitive to detecting low amounts of *P. neglectus* DNA isolated from water suspension and infected cereal roots (Fig. 2; Table 1).

**Figure 3:**
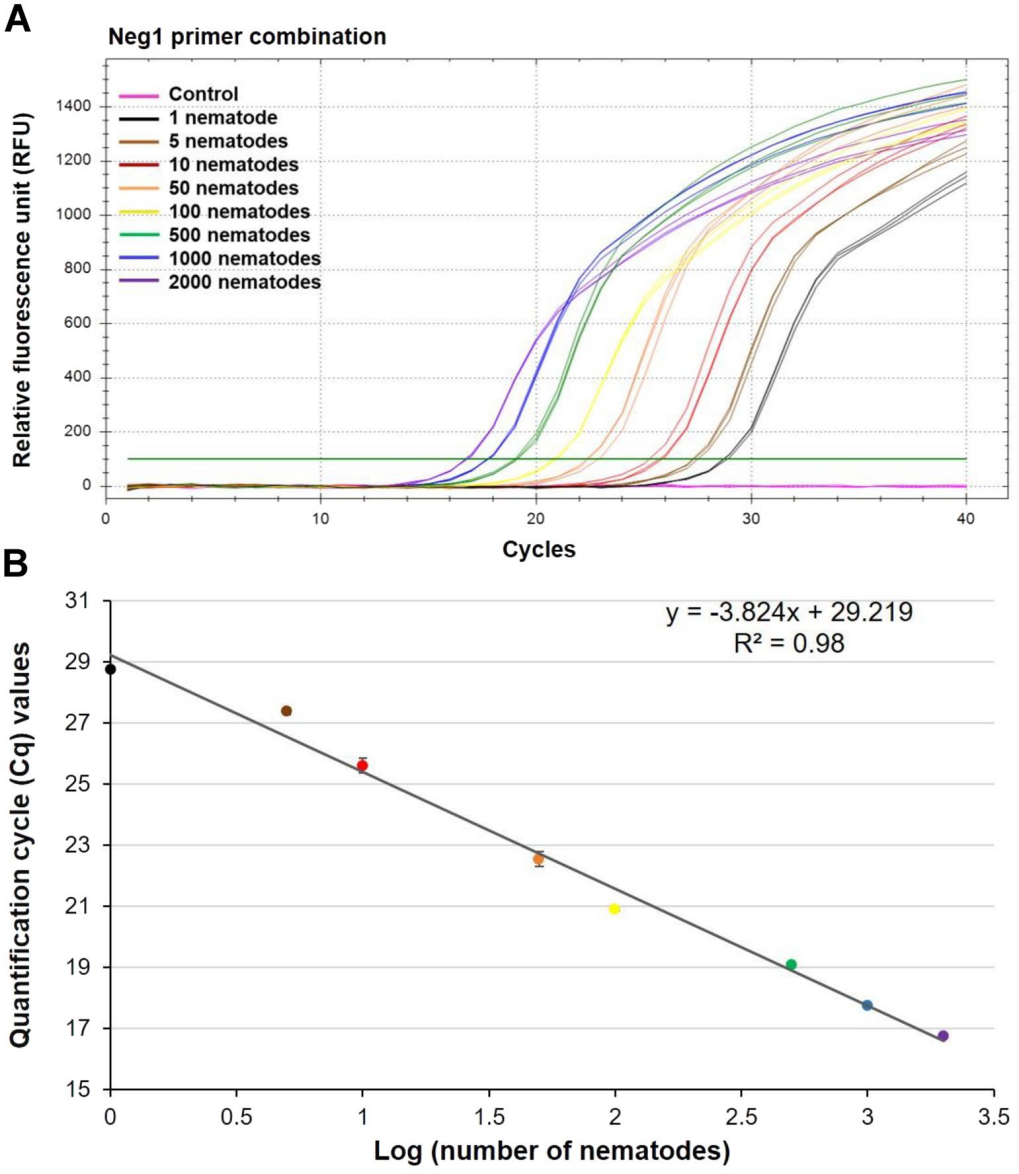
RT-qPCR experiments with *P. neglectus* DNA isolate PnGLS4 from varying numbers of nematodes. The Neg1 primer combination was used. (**A**) RT-qPCR amplification curves with DNA isolated from samples with different numbers of nematodes. (**B**) Regression curve with quantification cycle (Cq) values obtained after performing RT-qPCR with DNA isolated from varying numbers of nematodes. Each data point represents the mean of Cq values from three independent reactions.

Next, we wanted to determine the relationship between the number of nematodes and the abundance of nematode DNA measured by RT-qPCR. Between one and 2000 *P. neglectus* nematodes were collected under a stereo microscope, and the DNA of each sample was isolated (Table 2). After RT-qPCR, the Cq values ranged from 28.76 ± 0.08 to 16.74 ± 0.04 for one nematode to 2000 nematodes, respectively (Table 2). The clear and distinct amplification curves were obtained for each dilution (Fig. 3A; Supplementary Fig. 6), with an overall efficiency of 95% for RT-qPCR. The linear regression curve indicated a strong negative correlation between the Cq values and the number of nematodes (R^2^ = 0.98) (Fig. 3B). This demonstrates that the Neg1 primer combination and RT-qPCR assay are suitable for quantifying *P. neglectus*.

**Table 2:** Quantification cycle (Cq) values from nematode dilution experiments with different numbers of *Pratylenchus neglectus* isolate PnGLS4.

Number of nematodes	DNA concentration (ng/µl)	Cq ± SD
1	< 0.1 ng/µl	28.76 ± 0.08
5	< 0.1 ng/µl	27.36 ± 0.10
10	0.21	25.60 ± 0.25
50	1.13	22.51 ± 0.24
100	3.08	20.89 ± 0.05
500	14.31	19.06 ± 0.04
1000	27.14	17.74 ± 0.02
2000	48.11	16.74 ± 0.04

The nematode suspensions were counted under a stereo microscope, and then the DNA was extracted from 1, 5, 10, 50, 100, 500, 1000, and 2000 nematodes. DNA concentration was measured using a Qubit. The Neg1 primer combination was used. A concentration of 0.1 ng/µl was the lower detection limit for Qubit. SD was calculated from three technical replicates. The overall efficiency of *P. neglectus* was 95%.

*SD* standard deviation, calculated from three technical replicates.

### The RT-qPCR assay enables the detection of *Pratylenchus neglectus* in cereal roots

**Figure 4:**
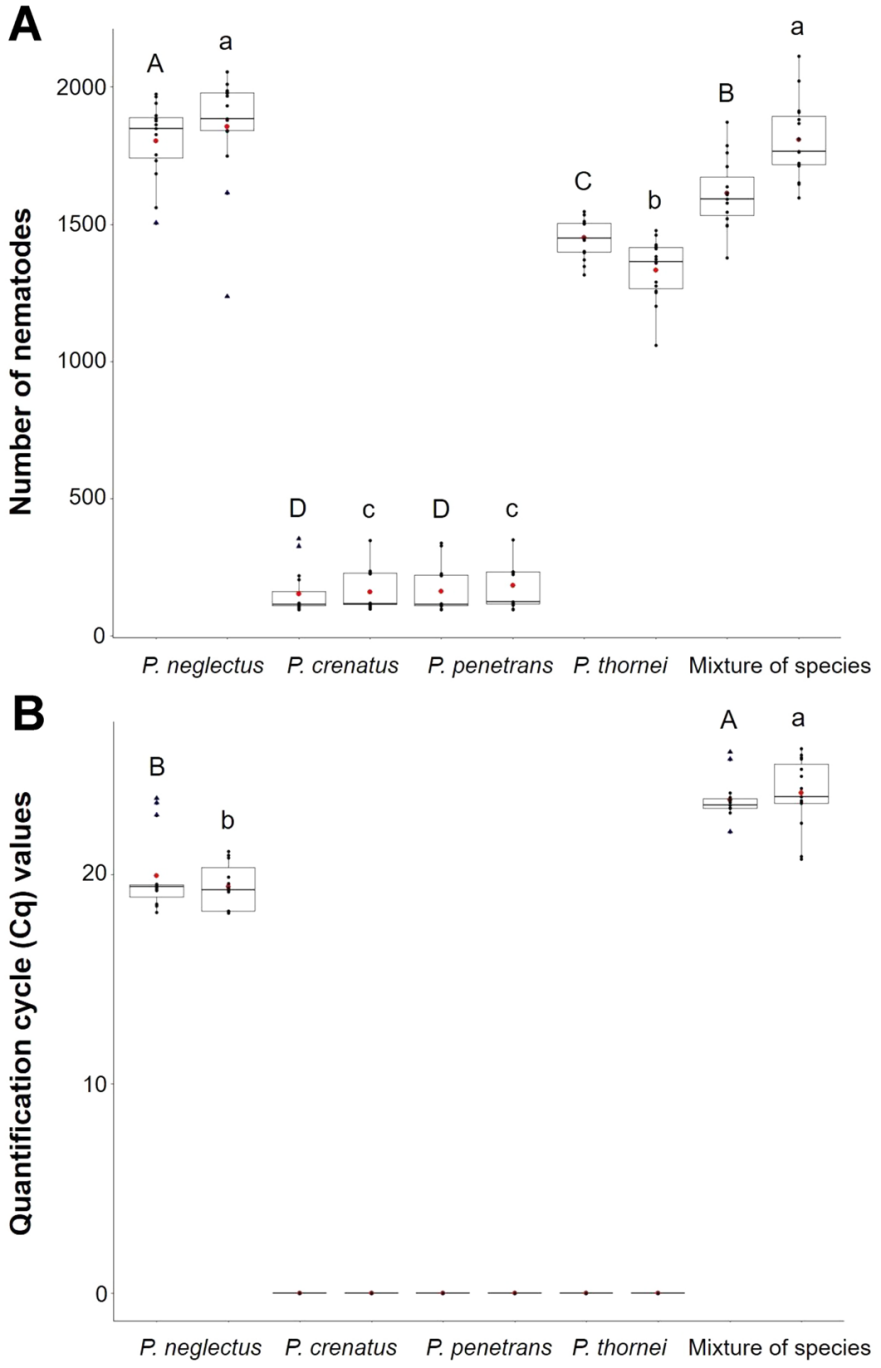
Inoculation experiments with different RLN species. Plants were inoculated with 1000 nematodes from different *Pratylenchus* species separately or as a mixture of all species (see Table 3). Roots were harvested eight weeks after inoculation and divided into two groups. DNA was isolated from one group, and nematodes were counted from the other. RT-qPCR was performed using the Neg1 primer combination. (**A**) The number of nematodes after visual counting; (**B**) quantification cycle (Cq) values. Individual and mean values are marked as black and red dots, respectively. The upper and lower quartiles are separated by the median (horizontal line). Blue triangles represent outliers. Error bars represent the standard error of the mean from biological replicates. An ANOVA test (p < 0.05) was performed, and significant differences between groups were calculated by a Tukey test (p < 0.05). Different letters (a–d), capital letters between barley plants, and small letters between wheat plants above error bars represent groups based on significance (see Table 3).

We performed two experiments (‘A’ and ‘B’) in the greenhouse to verify the specificity and sensitivity of the RT-qPCR assay for detecting and quantifying *P. neglectus* within infected roots. In experiment ‘A’, barley and wheat were infected with four different *Pratylenchus* species (*P. neglectus*, *P. crenatus*, *P. penetrans*, and *P. thornei*) separately or as a mixture of all species. Eight weeks after inoculation with 1000 nematodes, the physiological traits were measured (Supplementary Figs. 7 and 10). Then, the root samples were divided into two groups, one for nematode counting and the other for DNA isolation and RT-qPCR. The number of counted nematodes ranged between 161 ± 72 to 1856 ± 198 for barley and 154 ± 82 to 1804 ± 135 for wheat (Fig. 4A; Table 3). Most nematodes were found in treatments after infection with *P. neglectus* only and after infection with a mixture of species (Fig. 4A; Table 3), where a *P. neglectus*-specific primer combination also detected a DNA template of *P. neglectus* (Fig. 4B; Table 3). Interestingly, the infection rates between barley and wheat were almost similar after infection with *P. neglectus* only (barley 1.86 ± 0.20, wheat 1.80 ± 0.14) (Table 3). In contrast, barley and wheat are poor hosts for *P. crenatus* and *P. penetrans,* as indicated by the low final number of nematodes and Pf/Pi values (Fig. 4A; Table 3).

**Table 3:** Results of the DNA quantification experiments with cereal plants inoculated with different *Pratylenchus* species.

Plant species	Nematode inoculum	The final number of nematodes (Pf) ± SD	Pf/Pi ± SD	Cq value ± SD
Barley	Without nematode	N/A	–	N/A
Wheat		N/A	–	N/A
Barley	*P. neglectus*	1856 ± 198	1.86 ± 0.20	19.95 ± 1.74
Wheat		1804 ± 135	1.80 ± 0.14	19.42 ± 1.06
Barley	*P. crenatus*	161 ± 72	0.16 ± 0.08	N/A
Wheat		154 ± 82	0.15 ± 0.08	N/A
Barley	*P. penetrans*	184 ± 85	0.18 ± 0.08	N/A
Wheat		161 ± 82	0.16 ± 0.08	N/A
Barley	*P. thornei*	1333 ± 108	1.33 ± 0.11	N/A
Wheat		1452 ± 69	1.45 ± 0.07	N/A
Barley	*P. neglectus, P. crenatus, P. penetrans*, and *P. thornei*	1809 ± 138	1.81 ± 0.14	23.58 ± 0.92
Wheat		1612 ± 123	1.61 ± 0.12	23.90 ± 1.58

RT-qPCR was performed with DNA extracted from infected barley and wheat roots infected with 1000 nematodes per plant. Eight weeks after inoculation, roots were divided into two groups. Nematodes were extracted from one group using a misting chamber, followed by counting under a stereomicroscope. Pf/Pi is the multiplication ratio between the number of nematodes counted after harvesting (Pf) and the initial nematode inoculum (Pi). DNA was extracted from the second group, followed by RT-qPCR. The Neg1 primer combination was used for RT-qPCR. Each value represents an average of 15 single plants. The overall efficiency for barley and wheat were 86% and 83%, respectively.

*SD* standard deviation, *NA* not applicable.

Then, we compared the results from nematode counting and RT-qPCR data using Neg1, *P. neglectus*-specific primer combination. After infection with pure *P. neglectus* inoculum, the barley and wheat Cq values were 19.95 ± 1.74 and 19.42 ± 1.06, respectively. They were considerably lower after mixed infections with different nematode species (barley 23.58 ± 0.92; wheat 23.90 ± 1.58). As expected, the amplification curves obtained with DNA from roots not infected with *P. neglectus* did not cross the threshold line (Table 3). This confirmed the species specificity of the RT-qPCR detection assay (Fig. 4B; Table 3). It is also important to note that *P. neglectus* can be precisely identified after mixed infections, in contrast to tedious and time-consuming microscopy.

**Figure 5:**
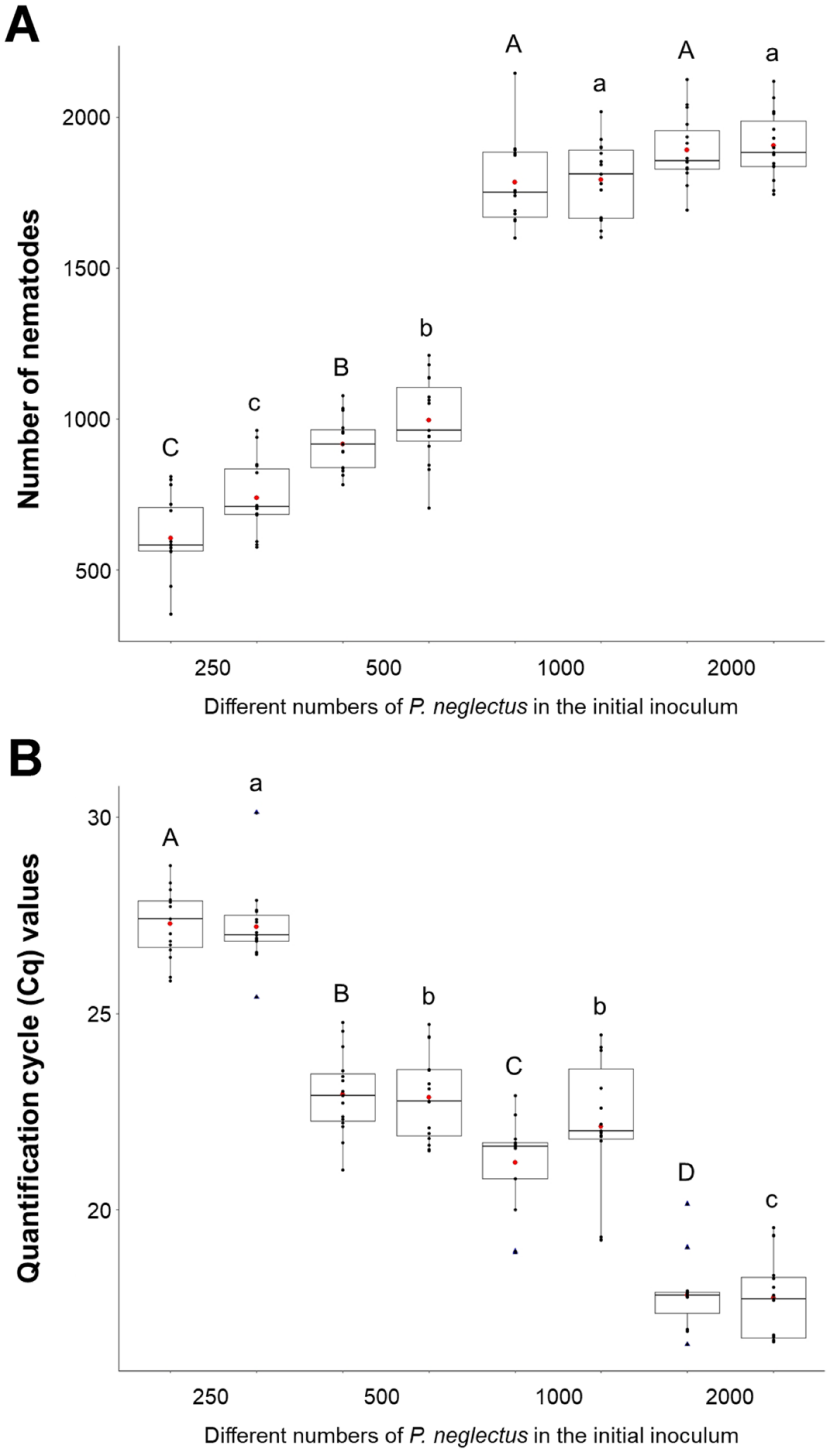
Inoculation experiments with varying numbers of *P. neglectus* isolate PnGLS4 to determine the correlation between nematode counting results and RT-qPCR (see Table 4). Plants were inoculated with different numbers of nematodes. Eight weeks after inoculation, root samples were divided into two groups. DNA was isolated from one group, and nematodes were counted from the other. RT-qPCR was performed using the Neg1 primer combination. (**A**) The number of nematodes counted under a stereo microscope, (**B**) quantification cycle (Cq) values after RT-qPCR. For experimental procedure and statistics, see this figure.

In the next experiment (‘B’), we determined the sensitivity of the DNA-based detection assay. We reasoned that the quantification cycle (Cq) values are correlated with the number of nematodes. Barley and wheat plants were infected with varying numbers of *P. neglectus,* and eight weeks after inoculation, the chlorophyll content, dry root, and shoot weight were measured. As a result, the increasing number of nematodes negatively impacted these parameters (Supplementary Figs. 8 and 10). After harvesting, half of the root samples were used for nematode counting and the other half for DNA isolation and RT-qPCR. The nematodes within the roots were counted under the microscope. The reproduction rates (Pf/Pi) decreased with the increasing number of nematodes in the inoculum (Table 4). After inoculation with 250 nematodes, the ratio ranged from 2.42 ± 0.52 to 2.95 ± 0.47 for wheat and barley, respectively, whereas after inoculation with 2000 nematodes, a much lower ratio was found (for both barley and wheat, 0.95 ± 0.05). Infection with 1000 and 2000 *P. neglectus* nematodes resulted in the highest final number of nematodes. After infection, the final number of nematodes with 1000 *P. neglectus* ranged between 1793 ± 122 and 1785 ± 139 for barley and wheat, respectively (Fig. 5A; Table 4). The Cq values ranged from 21.20 ± 1.10 to 22.13 ± 1.70 (Fig. 5B; Table 4). The number of nematodes after inoculation with 2000 nematodes was in the same range (barley 1905 ± 108, wheat 1891 ± 110) (Fig. 5A); however, the Cq values were strikingly lower (barley 17.82 ± 0.85, wheat 17.76 ± 1.02) (Fig. 5B; Table 4). Furthermore, the correlation between Cq values and the initial nematode inoculum was negative, with R^2^ ranging between 0.97 and 0.94 for barley and wheat, respectively (Supplementary Fig. 9), which could be explained by the fact that DNA from eggs and dead nematodes was amplified. At the same time, the visual counting method can only assess the number of viable nematodes.

**Table 4:** Results of DNA quantification experiments with different numbers of *P. neglectus* in the initial inoculum.

Number of nematodes in the inoculum (Pi)	Plant species	The final number of nematodes (Pf) ± SD	Pf/Pi ± SD	Cq value ± SD
Control	Barley	N/A	–	N/A
	Wheat	N/A	–	N/A
250	Barley	738 ± 116	2.95 ± 0.47	27.29 ± 0.85
	Wheat	604 ± 130	2.42 ± 0.52	27.21 ± 0.96
500	Barley	998 ± 138	1.99 ± 0.28	22.94 ± 1.01
	Wheat	915 ± 85	1.83 ± 0.17	22.87 ± 1.07
1000	Barley	1793 ± 122	1.79 ± 0.12	21.20 ± 1.10
	Wheat	1785 ± 139	1.78 ± 0.14	22.13 ± 1.70
2000	Barley	1905 ± 108	0.95 ± 0.05	17.82 ± 0.85
	Wheat	1891 ± 110	0.95 ± 0.05	17.76 ± 1.02

The quantification cycle (Cq) values were measured by RT-qPCR with DNA extracted from infected roots of inoculated barley and wheat plants. Ten days old plants were infected with different numbers of *P. neglectus* isolate PnGLS4. After 8 weeks, root samples were divided into two groups. In the first group, the nematodes were extracted from the roots using the misting chamber, followed by counting the number of nematodes using a stereomicroscope. Pf/Pi is the multiplication ratio between the number of nematodes counted after harvesting (Pf) and the initial nematode inoculum (Pi). DNA was extracted from the second group, followed by RT-qPCR. The Neg1 primer combination was used for RT-qPCR. Each data point represents an average of 15 single plants.

*SD* standard deviation, *NA* not applicable.

**Figure 6:**
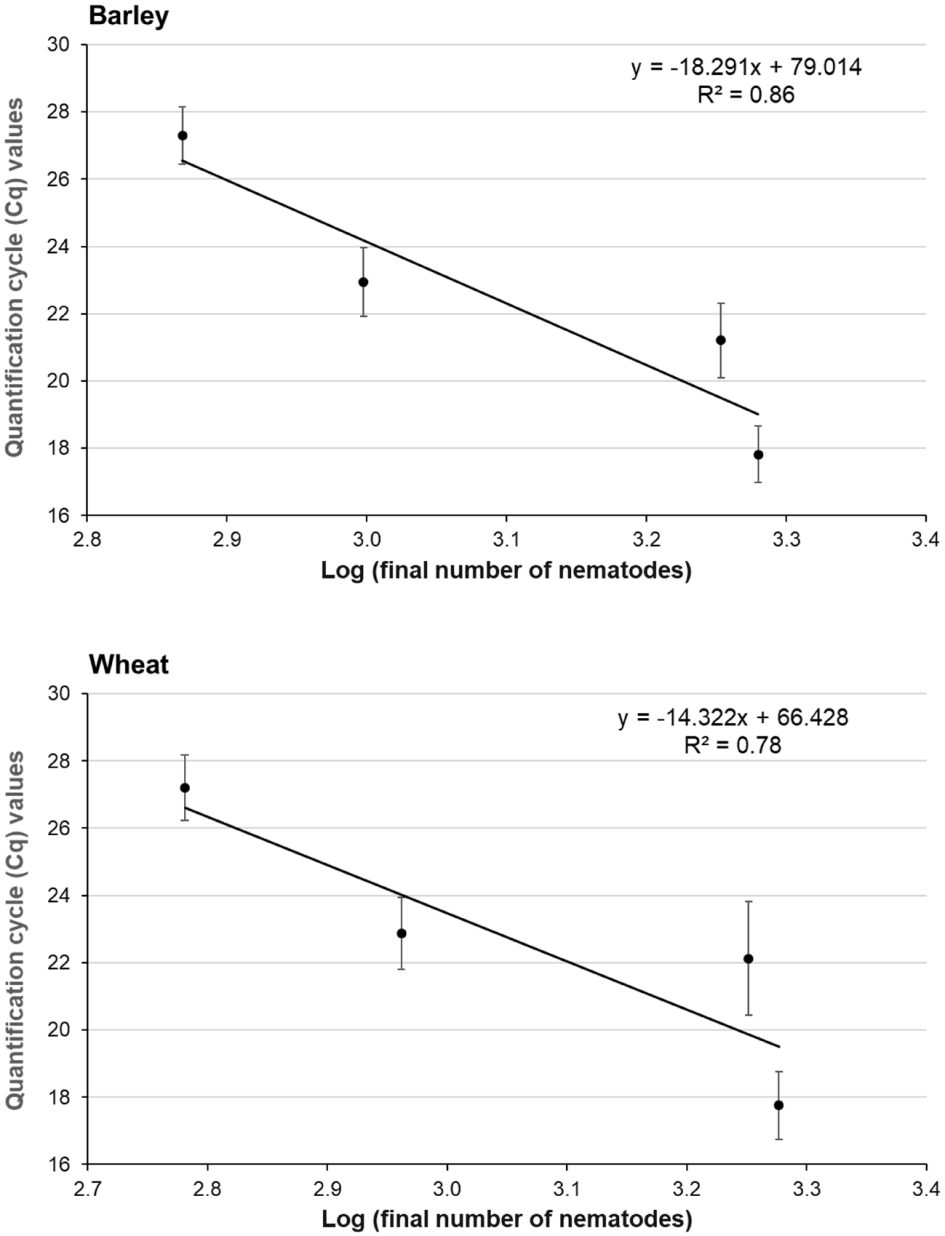
Regressions between the quantification cycle (Cq) values and the logarithm transferred of the final number of nematodes. Regressions were calculated with infection data from wheat and barley. The Neg1 primer combination was used for amplification. Each data point is the mean of fifteen biological and three technical repetitions. Plants were inoculated with different numbers of nematodes per plant (250, 500, 1000, and 2000) when they were 10 days old. Eight weeks after the infection, the roots were harvested (see Table 4).

We calculated the linear regression between the Cq values and the number of nematodes counted 8 weeks after inoculation, which showed a negative correlation *r*(2) = −0.93, *p* < 0.001 and *r*(2) = −0.88, *p* < 0.001 for barley and wheat, respectively. This allowed us to calculate the number of all nematodes at different stages of development within a root based on the Cq values (Fig. 6).

## Discussion

We have developed an RT-qPCR detection assay for detecting and quantifying *P. neglectus* in cereal roots. We utilized publicly available primer combinations information and RLN sequences from public databases to evaluate and identify the most effective *neglectus*-specific primer combination until the current study to avoid confusion in selecting the species-specific primer combination. Furthermore, we presented a protocol to extract the total DNA of the infected cereal roots. Moreover, we focused on a SYBR^®^ Green-based approach because it is affordable, fast, and accurate without reducing sensitivity. It is sensitive enough to detect the genomic DNA of a single nematode in a water suspension and to detect a minimum of ~ 250 nematodes within an infected root.

The total DNA sample represented different developmental stages of the nematode, such as juveniles, adults, eggs, and root DNA. Sato, et al.^40^ showed that the population density of *P. penetrans* in the soil evaluated by RT-qPCR may vary depending on the mixture of different life stages. They observed that *P. penetrans* Cq values might vary in the adult to juvenile stages. In contrast, Yan, et al.^35^ reported no significant difference in the Cq values between a single adult female, a single second-stage juvenile, and a single egg, demonstrating that different life stages of *P. neglectus* contain relatively equal amounts of DNA. Their results also suggest that the life stages of *P. neglectus* and the proportions of juveniles, adult females, and eggs in individual samples have no effect on RT-qPCR detection and quantification and are thus unlikely to explain the discrepancy between nematode levels measured by RT-qPCR and binocular counting. This follows the fact that, like most nematodes, *P. neglectus* has a fixed number of cells and nuclei established during early embryonic development which does not change throughout the life cycle^35^.

Among all species-specific primer combinations analyzed here with the SYBR^®^ Green method, the Neg1 primer combination provided the best performance and specific amplification from the infected root samples containing *P. neglectus* DNA templates in this study. Based on the bioinformatics, laboratory, and greenhouse investigations, this primer combination showed specificity, sensitivity, and lack of secondary structure. This primer combination binds within the internal transcribed spacer sequences of the ITS1 and 5.8S regions. The left primer binds to ITS1, whereas the right primer binds to the 5.8S region. Our findings, together with existing information regarding the Neg1 primer region and the sensitivity of detection of nematodes in soil samples, make Neg1 primer combination ideal for detecting and identifying *P. neglectus*. Furthermore, the linear regression curve for different numbers of nematodes in water suspension (1, 5, 10, 50, 100, 500, 1000, and 2000) demonstrated a strong negative correlation between the Cq values and the counted number of nematodes. Our assay’s sensitivity was comparable to that of previous RT-qPCR studies. However, these studies extracted DNA from nematodes in a water suspension or from the soil. *P. thornei* and *P. neglectus* populations could be quantified from soil samples^21,34,41^. In our study, a discrepancy between Cq values and the counted number of nematodes was found, which can be explained by the fact that eggs and inactive/dead nematodes can also be detected and quantified directly within the infected root. Thus, the sensitivity of the RT-qPCR detection assay is higher than the traditional visual counting method.

We also observed variations in nematode numbers that were counted under the microscope. These can be explained due to several factors, including a lack of misting chamber efficiency, which can only extract the active stages of the nematode, dilution from nematode synchronized suspension of nematode for visual counting, variation in the number of nematodes in different biological samples due to migratory lifestyle of nematode, and the effect of temperature on nematode reproduction. All traditional extracting methods, such as the misting chamber, Baermann funnel, and whitehead and hemming tray techniques^42,43^, rely on the active movement of juveniles and adults from moistened soil or cut tissue into the surrounding water. In case of a mixed infection, distinguishing between *Pratylenchus* species under the binocular microscope is challenging^44^. Furthermore, natural variation due to phenotypic plasticity^45^ and morphometric variation between and within species even more complicates morphological identification and quantification^17^.

RT-qPCR detects DNA from juveniles, adults, and eggs. Furthermore, all *Pratylenchus* spp. life stages can survive within the root and/or under arid conditions in an inactive, dehydrated, and dormant anhydrobiotic form^31^, which can be detected by RT-qPCR but may be difficult to detect by traditional methods. Assessing the number of nematodes in the soil is crucial for setting economic thresholds to initiate countermeasures like growing resistant varieties. The Economic damage threshold may vary significantly based on geographical location, climate, host tolerance, nematode virulence, the market value of the crop, and the cost of control measures^31^. For example, Castillo and Vovlas^7^ reported a wide range of *P. thornei* damage thresholds on wheat, ranging from 420 to 30,000 nematodes/kg of soil in Australia, France, and Mexico. Since the described RT-qPCR detection assay is an effective, fast, and low-cost method to estimate nematode numbers based on Cq values, we recommend a new method for estimating EDT using generated linear regression and its formula based on Cq values and/or calculated number of nematodes.

DNA-based pathogen and pest diagnostics have already become routine for massive screenings of soil samples for the abundance of plant parasitic and free-living nematodes. It is generally accepted that DNA-based diagnostics will gain more importance. Based on this study and available information, we recommend using the identified optimal primer combination and SYBR^®^ Green-based detection RT-qPCR assay for identifying and quantifying *P. neglectus* in water suspension, soil, and infected root. However, it provides a better understanding when combining morphological and molecular methods. Therefore, the approach should be assessed based on the purpose of the study, time, human source, and estimated budget. The detection assay presented here will be particularly interesting for plant breeding, where large populations must be screened quickly.

## Materials and methods

### Plant materials and growth conditions

The barley cultivar “Valentina”^46,47^ and the wheat cultivar “Machete”^48,49^ are both susceptible to *P. neglectus*. In experiment ‘A’, barley and wheat plants were inoculated with different *Pratylenchus* species, using 1000 nematodes per plant. In experiment ‘B’, barley and wheat plants were inoculated with an increasing number of a single *P. neglectus*. The greenhouse experiments were performed in 2021 and 2022 using the protocols described in Keil, et al.^47^.

Seeds were germinated on Whatman filter paper for two days in the dark at 26 °C. Then, seedlings were transplanted into plastic cylindrical tubes with a diameter of 4 cm and a height of 15 cm filled with heat-sterilized sand (Probau^®^ Quarzsand eco, grain size: 0.1–0.4 mm). Sieves with a mesh size of 20 µm pores were fixed at the bottom of the tubes to prevent loss of sand, root outgrowth, and nematode escape during the experiment. Plants were randomly arranged with 8 × 8 cm spacing between tubes. Plants were grown in the greenhouse under long-day conditions (16 h light) at 23 °C during the day and 18 °C at night with supplemental light (Son-T Agro 400W, Koninklijke Philips Electronics NV, Eindhoven, The Netherlands). Plants were irrigated twice a week from the bottom of the tubes with a nutrient solution, as described by Marshall and Ellis^50^. The nutrient solution was supplied from a 100-L tank and renewed monthly to avoid changes in nutrient concentrations. All experiments were performed in a completely randomized design.

### Nematode multiplication and greenhouse infection tests

The nematodes (Supplementary Table 1) were kindly provided by the Institute for Epidemiology and Pathogen Diagnostics, Julius Kühn-Institute, Braunschweig, Germany, and RLNs were multiplied and maintained on monoxenic cultures of carrot calli^51,52^. First, carrot discs were surface sterilized over a flame and incubated at 23 °C for one week. Next, nematodes were sterilized with streptomycin sulfate (10%), and 200 nematodes at different stages of development were placed on each disc. Then, each Petri dish containing one carrot disc was sealed with parafilm and stored in the dark at 25 °C. Every two weeks, the carrot disc cultures were checked for contaminations. Ninety days after inoculation, the nematodes were extracted from carrot calli for greenhouse infection tests. To prepare the initial inoculum, nematodes were counted in three 500 µl samples of nematode suspension. The total inoculum was adjusted with sterile water to a final concentration of 500 nematodes per ml. Then 2 ml nematode suspension was used to inoculate the plants. Finally, an equal number of four *Pratylenchus* species were visually counted and mixed to prepare the mixed nematode species inoculum.

Seedlings were grown for ten days in the greenhouse before nematode inoculation. After inoculation, the sand was covered with black plastic beads to avoid algal growth. Eight weeks after inoculation, plants were harvested. Dry shoot and root weights were measured, and the chlorophyll contents were determined by a Dualex instrument (Force A, Paris, France), according to Casa, et al.^53^. After that, the shoots were cut, half of the root samples were placed in a freeze dryer for DNA isolation and RT-qPCR, and the other half in a misting chamber to extract the nematodes for visual counting. The number of nematodes per plant was counted in one ml suspension three times using a stereomicroscope (magnification 32-fold). The total nematode numbers were calculated for the whole nematode suspension from each plant. Pf/Pi values were calculated as the ratio between the final numbers of nematodes at the end of the test (Pf) divided by the initial number of nematodes used for inoculating the plants (Pi).

### DNA extraction

A protocol for isolating total DNA from infected cereal roots, including nematode and plant DNA, was further modified^27^. After harvesting the plants eight weeks after inoculation, the whole root of each plant was freeze-dried and homogenized with a Geno/Grinder 2010 (SPEX@SamplePrep LLC, USA) for three minutes at 1000 strokes per minute. Then, five ml extraction buffer per gram of dry root was added. The mixture was incubated overnight at 56 °C in a water bath. One ml of the homogenized mixture was combined with one ml of chloroform/isoamyl alcohol (24:1) and phenol (1:1) in a fresh tube, shaken vigorously for 10 min, and centrifuged at 13,000 rpm for 10 min at room temperature (RT). Then, 700 µl of the supernatant was transferred to a new tube and combined with the same volume of chloroform/isoamyl alcohol (24:1), shaken for 10 min, and centrifuged for 10 min at 13,000 rpm at RT. This step was repeated twice, and finally, the DNA was precipitated by adding 400 µl ice-cold isopropanol at −20 °C overnight. The tubes were centrifuged at 4 °C for 15 min at 10,000 rpm, and the total DNA pellets were subsequently washed with 70% and 95% ethanol for five minutes. After drying at RT, the total DNA was resolved in 200 µl low TE. The quality of the total DNA was checked by gel electrophoresis and quantified with a Qubit™ 4 Fluorometer (Invitrogen by ThermoFisher Scientific, Singapore). Before RT-qPCR, the total DNA was diluted to a final concentration of 10 ng/µl.

Nematode DNA was extracted with slight modifications using the protocol from Al-Banna et al. (2004). First, a distinct number of nematodes obtained after counting was collected in 100 µl distilled water and freeze-thawed thrice for 30 min at −80 °C. Then, 0.2 ml extraction buffer with 2 µl Proteinase K (Biotechrabbit™) was added, and the lysed nematodes were kept overnight at 56 °C in a water bath. The next day, chloroform/isoamyl alcohol (24:1) was added following shaking, the phases were separated, and the DNA was precipitated with one volume of cold isopropanol at −20 °C. Finally, the DNA pellets were washed twice with 70% ethanol and resolved in low TE.

### PCR and real-time quantitative PCR

We searched the literature and the National Center for Biotechnology Information (NCBI) database for *P. neglectus*-specific primer combinations. We found four primer combinations which had been used for PCR and RT-qPCR studies (Supplementary Table 2). Since this study aimed to develop a fast, affordable, and precise detection assay, the focus was on using the SYBR^®^ Green method. PCR was carried out with a Life Touch Thermal cycler (TC-96, Hangzhou Bioer Technology Co., LTD. China) in a 20 µl volume containing the DNA template (2 µl), 0.1 µl of Taq-polymerase (Biozym Scientific GmbH), 0.4 µl of 10 mM dNTPs, 0.3 µl of 10 pM of each primer, and 2 µl of 10× PCR-buffer (Biozym Scientific GmbH). Five µl of the PCR products were analyzed by electrophoresis in 3% agarose gels. Amplification occurred in a thermal cycler using the following program: 5 min at 94 °C as initial denaturation; followed by 35 cycles of 30 s at 94 °C for denaturation; 30 s at a specific annealing temperature of each primer combination (Supplementary Table 2), 30 s at 72 °C for extension, and final extension at 72 °C for 5 min. In addition, the Bio-Rad CFX Connect^TM^Optics Module Real-time PCR detection system was used for RT-qPCR (Bio-Rad Laboratories, Inc., Singapore). Therefore, 10 µl of Platinum^®^ SYBR^®^ Green (qPCR SuperMix-UDG with ROX) (Invitrogen) were mixed with five µl of normalized ten ng/µl DNA solution, one µl primer solution (10 pM), and 3 µl distilled water. The thermal cycle was programmed for 3 min at 95 °C as initial denaturation, followed by 40 cycles of 10 s at 95 °C for denaturation, 30 s at a specific annealing temperature of each primer combination (Supplementary Table 2), 30 s at 72 °C for extension, and final extension at 95 °C for 10 s. To confirm the flanked sequence with each primer combination, PCR products from specific primer combinations for *P. neglectus* were sequenced using Sanger sequencing and CLC Main Workbench version 23.0.3 (CLC bio, Aarhus, Denmark).

### Bioinformatics and primer design

The Primer3 program (version 4.1.0) was used for validation length, melting temperature, GC content, and other PCR amplification characteristics, such as optimizing primer design based on specific PCR conditions such as annealing temperature, MgCl_2_ concentration, and template DNA concentration^54,55^. Beacon Designer™ Free Edition and the mFold software were used to predict secondary structures, their melting temperatures, the stability of DNA duplexes, and the potential for DNA binding interactions^55^. The primer sequences were BLASTed against the NCBI nucleotide database and the barley reference genome (http://www.ncbi.nlm.nih.gov/) to identify putative non-specific binding sites^55^.

### Statistical analysis

RT-qPCR data were analyzed with the Bio-Rad CFX Manager™ software version 3.1. The amplification efficiency (E) was calculated from the slope of a plot of the quantification cycle (Cq) (y-axis) and log picograms (log pg) of DNA (x-axis) using the equation E = (10^(1/–m)^ – 1) × 100, where *m* is the slope^56^. ANOVA was performed with the “Agricolae” program package in R Studio software, version 4.1.0. and significant differences between groups were calculated by a Tukey test (p < 0.05).

### Supplementary Information

Supplementary Information.

## Data Availability

The authors declare that data supporting the finding of this study are available from this manuscript and its supplementary information files. Extra data, information, and materials used in this study are available from the corresponding authors upon request. All methods were carried out in accordance with relevant guidelines. The sequence data obtained in this study are openly available in GenBank of NCBI at https://www.ncbi.nlm.nih.gov/ under Accession No. OR050567, KM593901, OR052247, and OR052248.

## References

[1] Decraemer, W. & Geraert, E. *Plant Nematology* (eds. Perry, R.N. & Moens, M.). 179–216 (2013).

[2] Bernard, G. C., Egnin, M. & Bonsi, C. *Nematology Concepts, Characteristics and Control* (eds. Shah, M.M. & Mahamood, M.) Chap. 7. 121–151 (InTech, 2017).

[3] Malik IM, Tak H, Lone GM, Dass WM (2022). Phytoparasitic nematodes as the major threat to viticulture. Environ. Exp. Biol..

[4] Elling AA (2013). Major emerging problems with minor *meloidogyne* species. Phytopathology.

[5] Singh S, Singh B, Singh AP (2015). Nematodes: A threat to sustainability of agriculture. Proc. Environ. Sci..

[6] Khanna, K. *et al.* Sustainable management of nematodes in agriculture, Vol.1: Organic management. in *Sustainability in Plant and Crop Protection* (eds. Chaudhary, K.K. & Meghvansi, M.K.). Vol. 157–185 (Springer Cham, 2022).

[7] Castillo, P. & Vovlas, N. *Pratylenchus (Nematoda: Pratylenchidae): Diagnosis, Biology, Pathogenicity and Management)*. Vol. 6 (Brill, 2007).

[8] Jones JT (2013). Top 10 plant-parasitic nematodes in molecular plant pathology. Mol. Plant Pathol..

[9] Smiley, R. W. & Nicol, J. M. *Wheat Science and Trade* (ed. Carver, B.F.). 171–187 (Wiley-Blackwell, 2009).

[10] Vanstone VA, Hollaway GJ, Stirling GR (2008). Managing nematode pests in the southern and western regions of the Australian cereal industry: Continuing progress in a challenging environment. Australas Plant Pathol..

[11] Smiley RW (2009). Root-lesion nematodes reduce yield of intolerant wheat and barley. Agron. J..

[12] Smiley RW (2015). Root-Lesion Nematodes: Biology and Management in Pacific Northwest Wheat Cropping Systems.

[13] Mokrini F, Viaene N, Waeyenberge L, Dababat AA, Moens M (2019). Root-lesion nematodes in cereal fields: Importance, distribution, identification, and management strategies. J. Plant Dis. Prot..

[14] Sharma S (2011). QTL analysis of root-lesion nematode resistance in barley: 1. *Pratylenchus** neglectus*. Theor. Appl. Genet..

[15] Techen AK, Helming K (2017). Pressures on soil functions from soil management in Germany. A foresight review. Agron. Sustain Dev..

[16] Hallmann J, Frankenberg A, Paffrath A, Schmidt HS (2007). Occurrence and importance of plant-parasitic nematodes in organic farming in Germany. Nematology.

[17] Nisa RU, Tantray AY, Shah AA (2022). Shift from morphological to recent advanced molecular approaches for the identification of nematodes. Genomics.

[18] Geraert, E. *The Pratylenchidae of the World: Identification of the Family Pratylenchidae (Nematoda: Tylenchida)*. (Academia Press, 2013).

[19] Tandonnet S, Pires-da Silva A (2016). Phenotypic plasticity and developmental innovations in nematodes. Curr. Opin. Genet. Dev..

[20] de Oliveira CMG, Monteiro AR, Blok VC (2011). Morphological and molecular diagnostics for plant-parasitic nematodes: Working together to get the identification done. Trop. Plant Pathol..

[21] Ophel-Keller K, Mckay A, Hartley D, Curran J (2008). Development of a routine DNA-based testing service for soilborne diseases in Australia. Australas Plant Pathol..

[22] Riley IT, Nobbs JM, McKay H, McKay AC (2009). *Pratylenchus* species in pastures in the South East Region of South Australia. Australas Plant Dis..

[23] Handayani ND (2020). Distribution, DNA barcoding and genetic diversity of potato cyst nematodes in Indonesia. Eur. J. Plant Pathol..

[24] Sanchez-Monge A (2017). *mtCOI* successfully diagnoses the four main plant-parasitic *Aphelenchoides* species (Nematoda: Aphelenchoididae) and supports a multiple origin of plant-parasitism in this paraphyletic genus. Eur. J. Plant Pathol..

[25] Avo AP (2017). DNA barcoding and morphological identification of benthic nematodes assemblages of estuarine intertidal sediments: Advances in molecular tools for biodiversity assessment. Front. Mar. Sci..

[26] Boroş L (2018). Detection and characterization of root-knot nematodes (*Meloidogyne* spp.) associated with three host plants in Romania. Roman. Biotechnol. Lett..

[27] Al-Banna L, Ploeg AT, Williamson VM, Kaloshian I (2004). Discrimination of six *Pratylenchus* species using PCR and species-specific primers. J. Nematol..

[28] Yan GP (2008). Detection and discrimination of *Pratylenchus** neglectus* and *P. **thornei* in DNA extracts from soil. Plant Dis..

[29] Qiu J, Westerdahl BB, Williamson VM (2007). Detection and quantification of root-lesion nematode *Pratylenchus*
*vulnus* using real-time PCR. J. Nematol..

[30] Hodson AK, Cicchetto A, Fierro FA (2021). Real time PCR assays to detect and quantify the nematodes *Pratylenchus*
*vulnus* and *Mesocriconema*
*xenoplax*. Crop Prot..

[31] Carrasco-Ballesteros S, Castillo P, Adams BJ, Perez-Artes E (2007). Identification of *Pratylenchus*
*thornei**,* the cereal and legume root-lesion nematode, based on SCAR-PCR and satellite DNA. Eur. J. Plant Pathol..

[32] Berry SD, Fargette M, Spaull VW, Morand S, Cadet P (2008). Detection and quantification of root-knot nematode (*Meloidogyne*
*javanica*), lesion nematode (*Pratylenchus*
*zeae*) and dagger nematode (*Xiphinema*
*elongatum*) parasites of sugarcane using real-time PCR. Mol. Cell Probe.

[33] Sato E, Goto K, Min YY, Toyota K, Suzuki C (2010). Quantitative detection of *Pratylenchus*
*penetrans* from soil using soil compaction and real-time PCR. Nematol. Res..

[34] Yan GP, Smiley RW, Okubara PA (2012). Detection and quantification of *Pratylenchus*
*thornei* in DNA extracted from soil using real-time PCR. Phytopathology.

[35] Yan G, Smiley RW, Okubara PA, Skantar AM, Reardon CL (2013). Developing a real-time PCR assay for detection and quantification of *Pratylenchus*
*neglectus* in soil. Plant Dis..

[36] Peetz AB, Zasada IA (2016). Species-specific diagnostics using a *β**-1,4-endoglucanase* gene for *Pratylenchus* spp. occurring in the Pacific Northwest of North America. Nematology.

[37] Jayatilake DV (2013). Genetic mapping and marker development for resistance of wheat against the root lesion nematode *Pratylenchus*
*neglectus*. BMC Plant Biol..

[38] Oliveira CMG, Blok V, Neilson R, Mroz T, Roberts D (2017). Hydrolysis probe-based PCR for detection of *Pratylenchus*
*crenatus*, *P.*
*neglectus* and *P.*
*penetrans*. Nematology.

[39] Lin BR, Tao Y, Wang HH, Liao JL, Zhuo K (2020). Duplex real-time quantitative PCR for simultaneous detection and quantification of *Pratylenchus*
*neglectus* and *P.*
*thornei*. Eur. J. Plant Pathol..

[40] Sato E, Min YY, Shirakashi T, Wada S, Toyota K (2007). Detection of the root-lesion nematode, *Pratylenchus*
*penetrans* (Cobb), in a nematode community using real-time PCR. Jpn. J. Nematol..

[41] Smiley RW (2013). Effects of crop rotations and tillage on *Pratylenchus* spp. in the semiarid Pacific Northwest United States. Plant Dis..

[42] Bezooijen JV (2006). Methods and Techniques for Nematology 118.

[43] EPPO, P. (ed. European and Mediterranean Plant Protection Organization). Vol. 43. 471–495 (EPPO Bulletin, 2013).

[44] Vogler AP, Monaghan MT (2007). Recent advances in DNA taxonomy. J. Zool. Syst. Evol. Res..

[45] Sommer RJ (2017). The genetics of phenotypic plasticity in nematode feeding structures. Open Biol..

[46] Galal A (2014). Comparative QTL analysis of root lesion nematode resistance in barley. Theor. Appl. Genet..

[47] Keil T, Laubach E, Sharma S, Jung C (2009). Screening for resistance in the primary and secondary gene pool of barley against the root-lesion nematode *Pratylenchus*
*neglectus*. Plant Breed..

[48] Taylor, S. P., Hollaway, G. J. & Hunt, C. H. Effect of field crops on population densities of *Pratylenchus**neglectus* and *P.**thornei* in southeastern Australia; part 1: *P.**neglectus*. *J.**Nematol.* **32**, 591–599 (2000). PMC2620495 19271014

[49] Williams J (2002). Mapping of the root lesion nematode (*Pratylenchus*
*neglectus*) resistance gene *Rlnn1* in wheat. Theor. Appl. Genet..

[50] Marshall B, Ellis RP (1998). Growth, yield and grain quality of barley (*Hordeum*
*vulgare* L.) in response to nitrogen uptake—I. A low cost, controlled nutrient supply system. J. Exp. Bot..

[51] Moody EH, Lownsbery BF, Ahmed JM (1973). Culture of the root-lesion nematode *Pratylenchus*
*vulnus* on carrot discs. J. Nematol..

[52] Kagoda F, Coyne D, Mbiru E, Derera J, Tongoona P (2010). Monoxenic culture of *Pratylenchus*
*zeae* on carrot discs. Nematol. Mediterr..

[53] Casa R, Castaldi F, Pascucci S, Pignatti S (2015). Chlorophyll estimation in field crops: An assessment of handheld leaf meters and spectral reflectance measurements. J. Agric. Sci.-Camb..

[54] Rozen S, Skaletsky H (2000). Primer3 on the WWW for general users and for biologist programmers. Methods Mol. Biol..

[55] Thornton B, Basu C (2011). Real-time PCR (qPCR) primer design using free online software. Biochem. Mol. Biol. Educ..

[56] Ginzinger DG (2002). Gene quantification using real-time quantitative PCR: An emerging technology hits the mainstream. Exp. Hematol..

