# Supplementary material for: DNA-based assessment of root lesion nematode infections in cereal roots
Source: Sci Rep. 2023 Aug 3;13:12602. doi: 10.1038/s41598-023-39559-8 (PMC10400682; doi:10.1038/s41598-023-39559-8)
Supplement: Supplementary file 1 — Supplementary Information. [file 41598_2023_39559_MOESM1_ESM.pdf]

DNA-based assessment of root lesion nematode infections in cereal roots

Ehsan Fatemi[1], Siegbert Melzer[1] and Christian Jung[1*]

[1]Plant Breeding Institute, Christian-Albrechts-University of Kiel, Olshausenstr. 40, D-24098 Kiel, Germany

[*]Corresponding author email: c.jung@plantbreeding.uni-kiel.de

Supplementary data

Supplementary figures

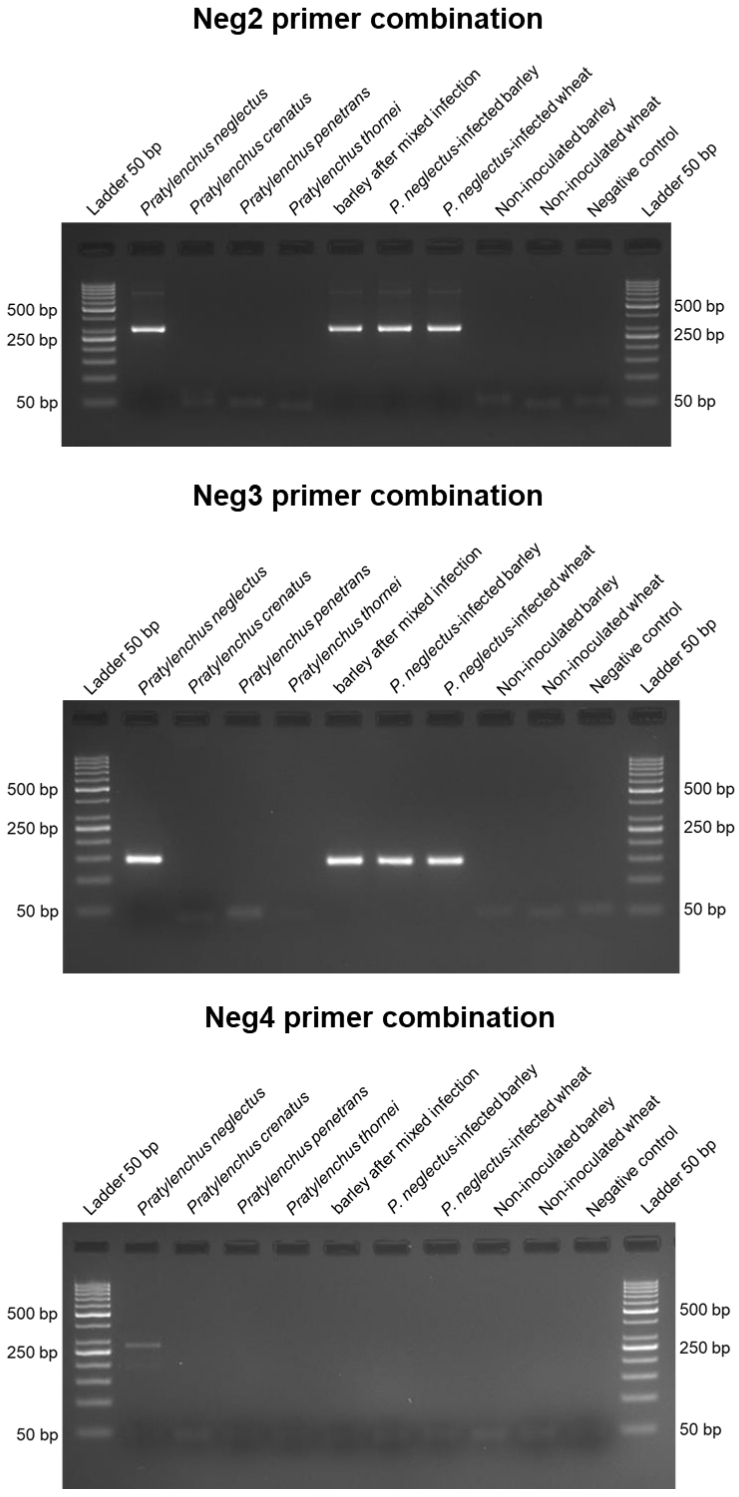

Supplementary Figure 1: Agarose gel electrophoresis with PCR fragments amplified from RLN DNA using different species-specific primer combinations. The primer sequences, PCR conditions annealing temperatures, and expected fragment sizes are given in the material and method and Supplementary Table 2. PCR fragments were separated in 3% agarose gels (80V for 60 minutes).

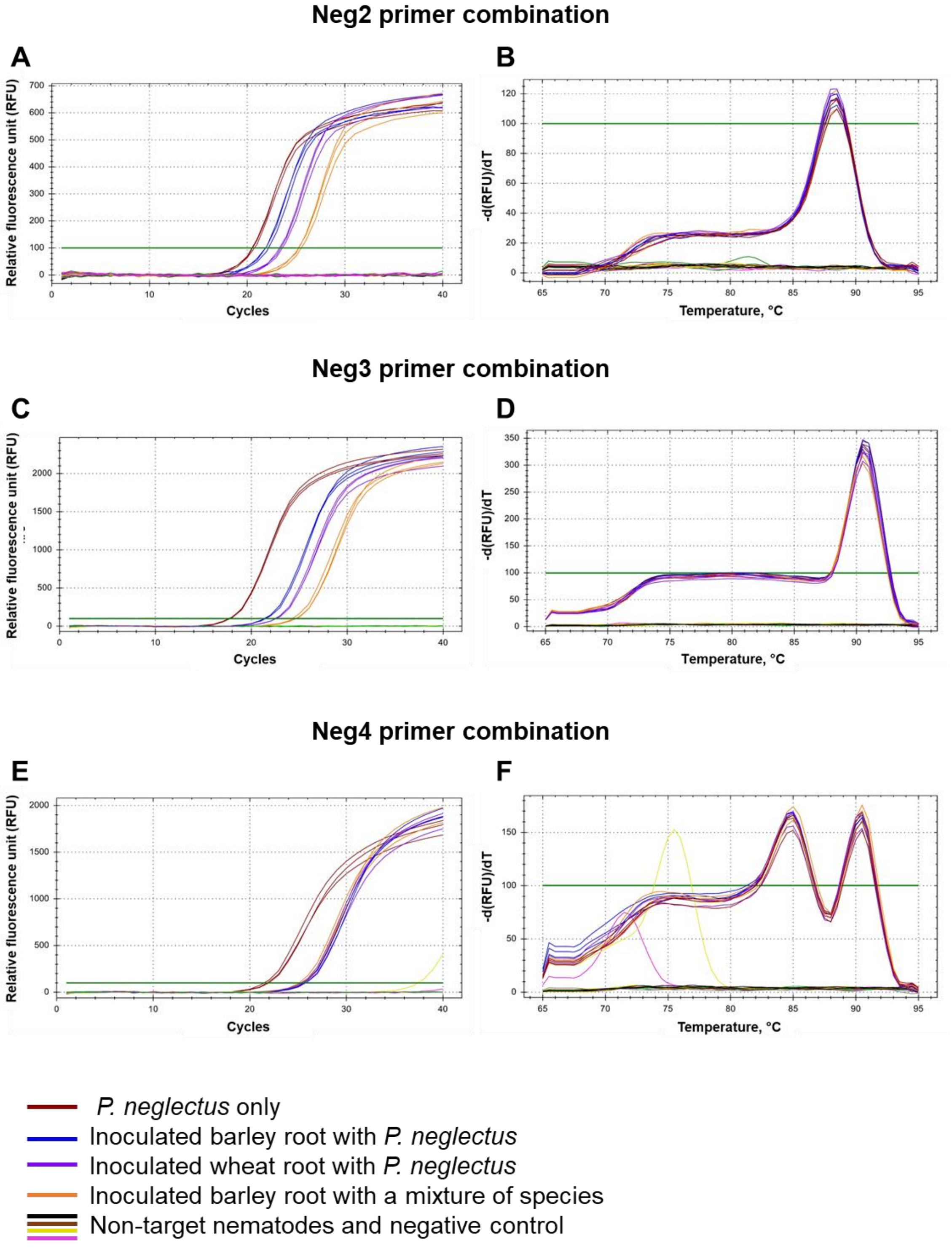

Supplementary Figure 2: Real-time quantitative polymerase chain reactions with species-specific primer combinations. The primer sequences, RT-qPCR conditions annealing, and melting temperatures are given in the material and method and Supplementary Table 2. All samples were analyzed using three technical replicates.

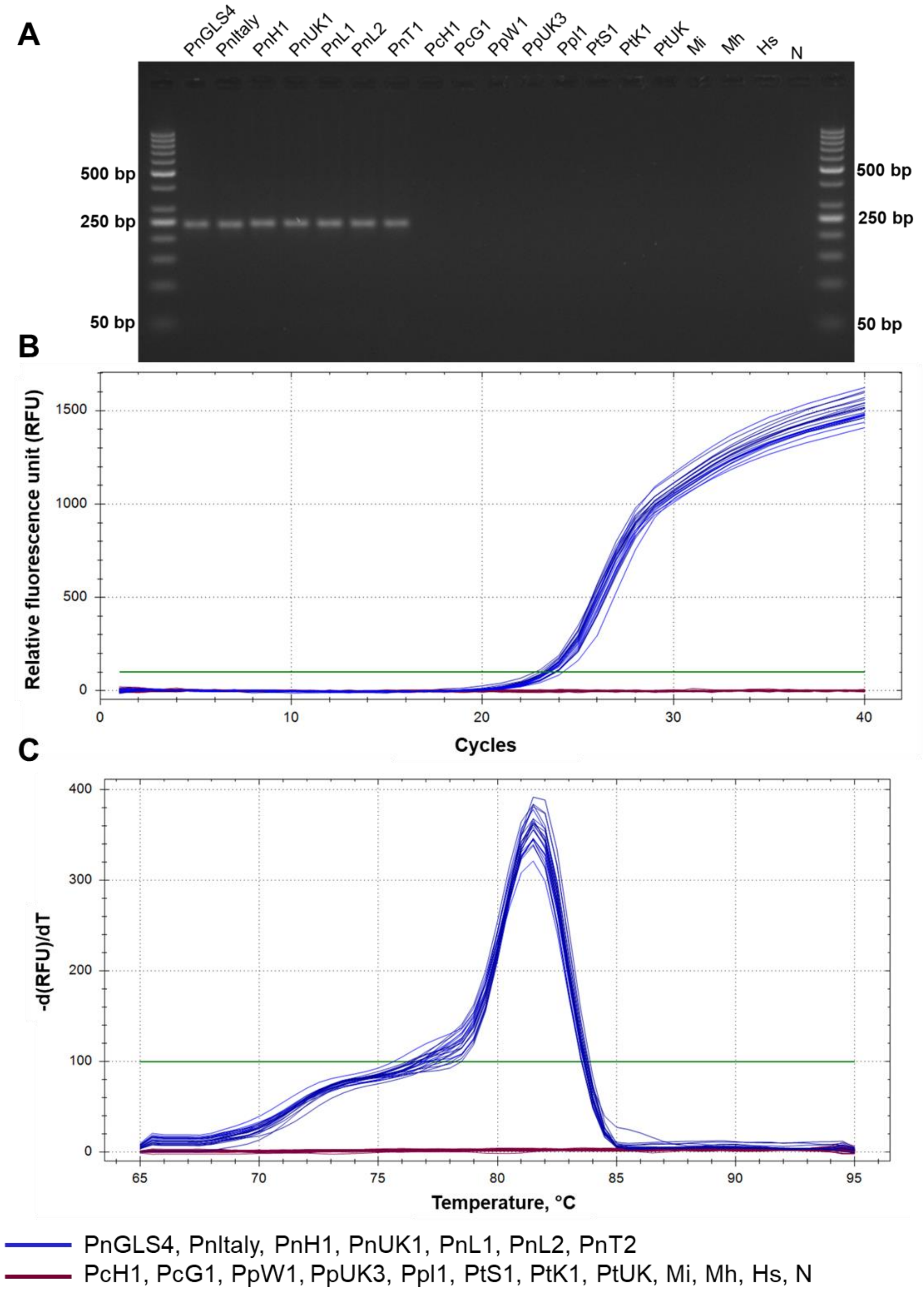

Supplementary Figure 3: PCR and RT-qPCR results with PPN DNA using primer combination Neg1. The primer sequences, PCR conditions annealing temperatures, and expected fragment sizes are given in the material and method and Supplementary Table 2. (A) Agarose gel electrophoresis (3%, 80V for 60 minutes) with PCR fragments amplified with DNA from different PPN species. (B) RT-qPCR amplification curves with DNA from seven *Pratylenchus neglectus* isolations and other PPNs (Supplementary Table 1). (C) The melting curve peak of the *P. neglectus*-specific amplicons is at 81.5°C. All samples were analyzed using three technical replicates. None of the non-*P. neglectus* amplification curves touch the threshold.

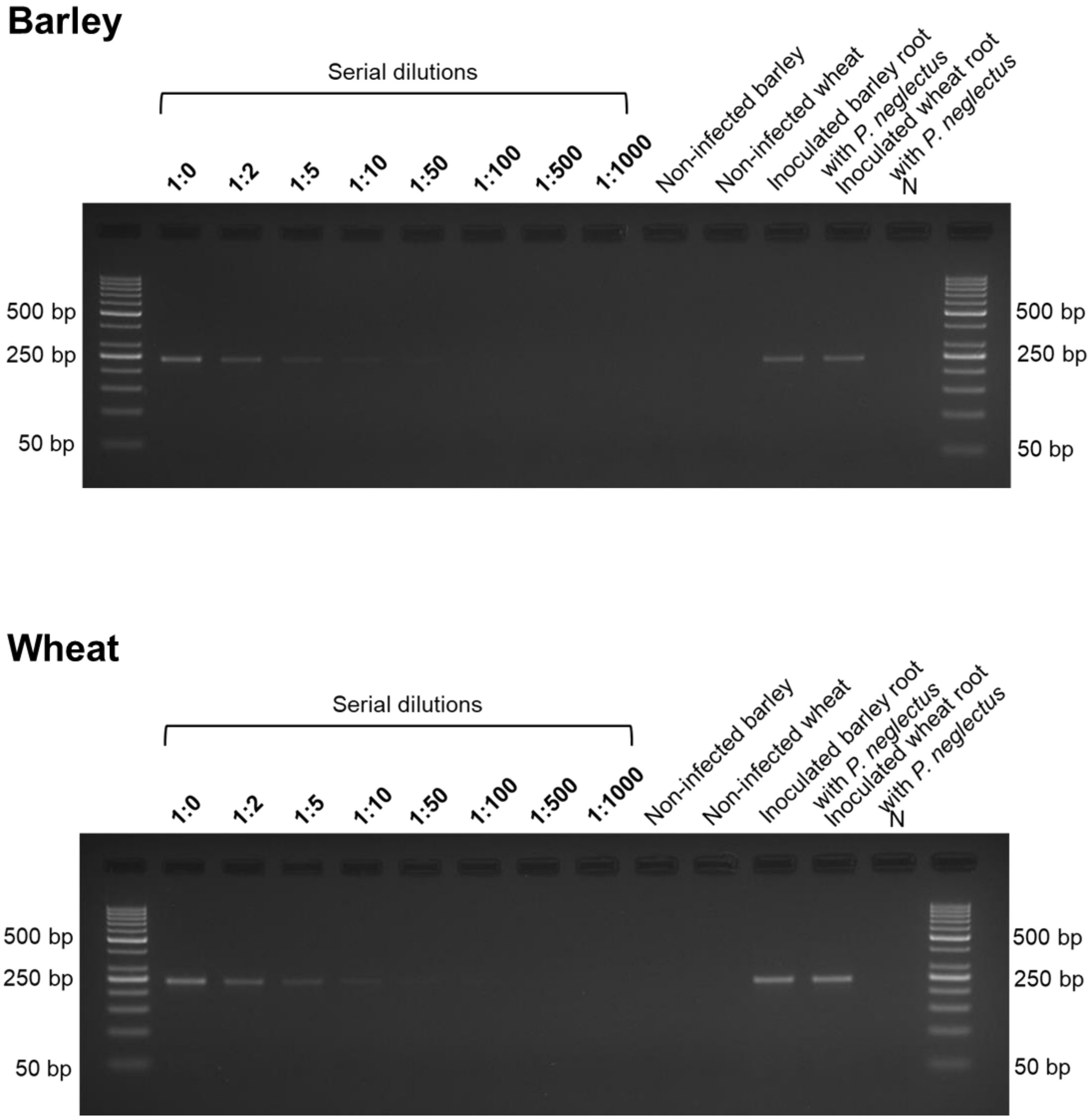

Supplementary Figure 4: PCR results from serial dilutions of total DNA from *P. neglectus* infected roots isolate PnGLS4. Total DNA was extracted from infected roots and amplified using the Neg1 primer combination. PCR conditions and expected fragment sizes are given in the material and method and Supplementary Table 2. DNA was separated in a 3% agarose gel (80V for 60 minutes).

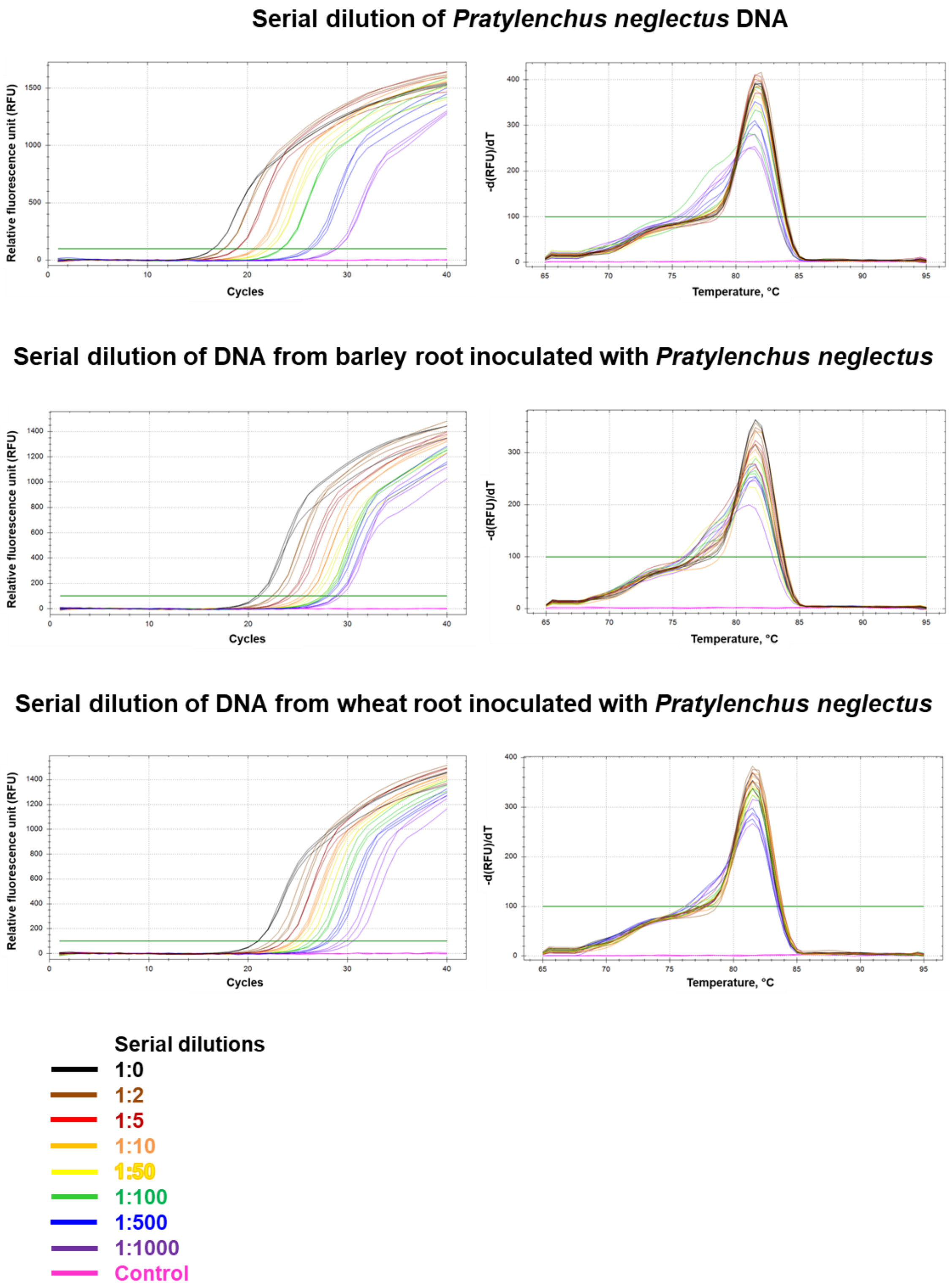

Supplementary Figure 5: Real-time quantitative polymerase chain reaction and melting curve profiles for serial dilutions of *P. neglectus* DNA and total DNA from barley and wheat roots infected with *P. neglectus*. Left: RT-qPCR amplification curves, Right: Melting peak of the samples at 81.5°C. The Neg1 primer combination was used for amplification. All samples were analyzed using three technical replicates; each Cq value is the mean of three technical replicates.

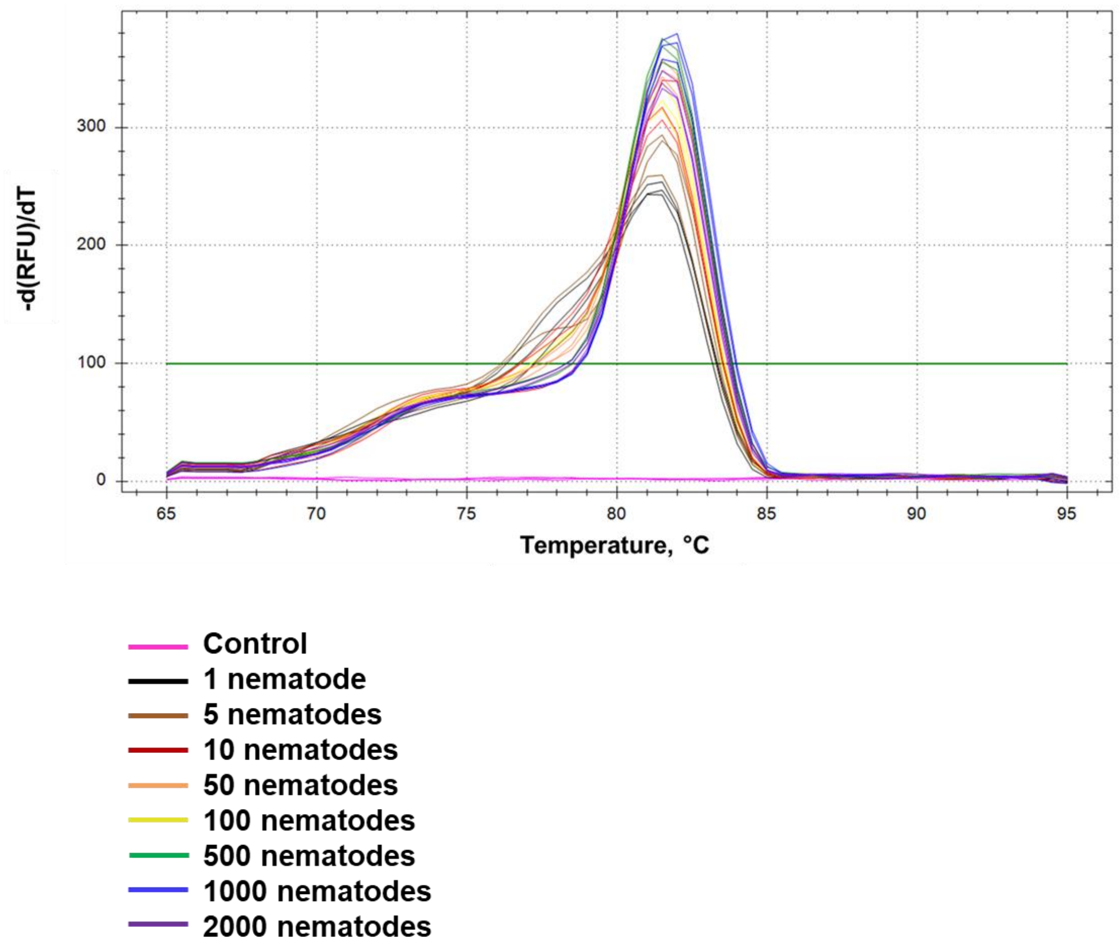

Supplementary Figure 6: Melting peak curves after RT-qPCR with *P. neglectus* DNA isolated from varying numbers of nematodes. The Neg1 primer combination was used. Curves for each sample show three technical replicates.

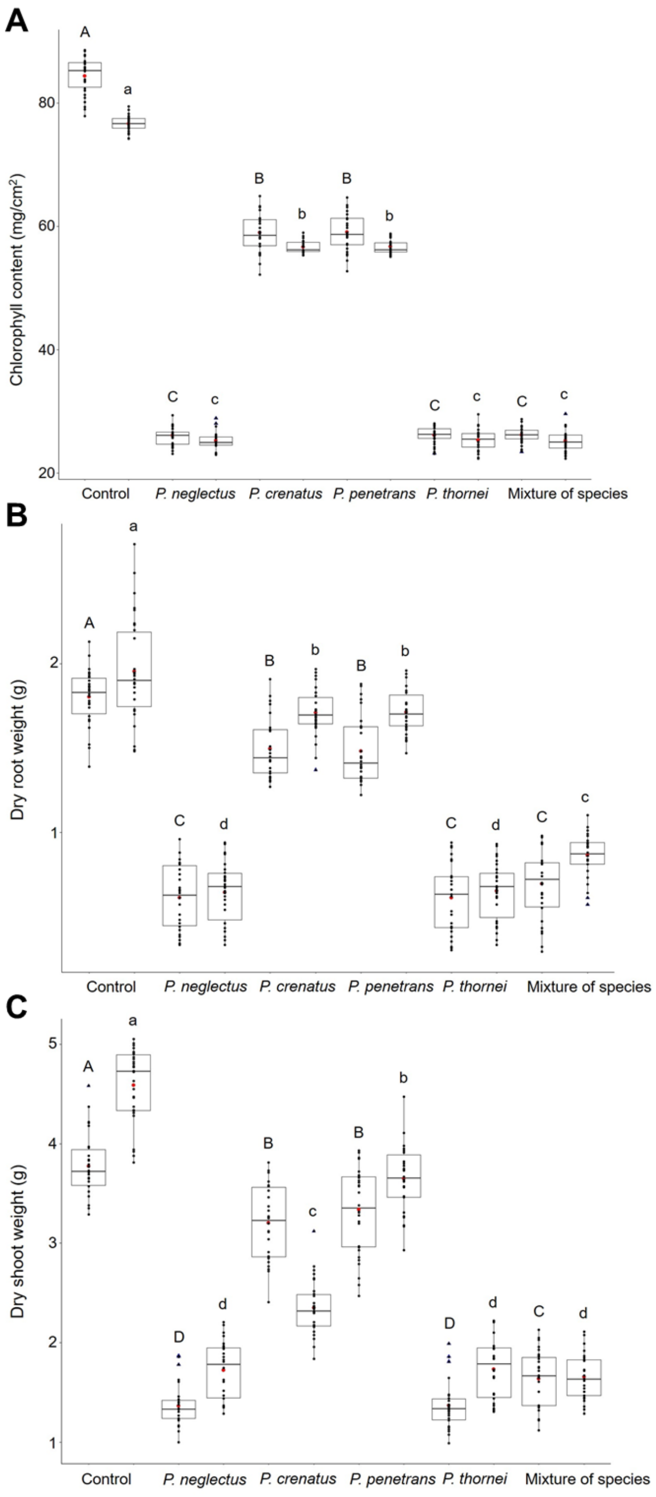

Supplementary Figure 7: Different RLN species affect plant growth and physiological traits. Plants were inoculated with 1000 nematodes from different *Pratylenchus* species separately or as a mixture of all species (Experiment 'A'). Roots were harvested eight weeks after inoculation. (A): Chlorophyll content, (B): Dry root weight, (C): Dry shoot weight. Individual and mean values are marked in black and red dots, respectively. The Upper and lower quartiles are separated by the median (horizontal line). Blue triangles represent outliers. Error bars represent the standard error of the mean from biological replicates. An ANOVA test ($p < 0.05$) was performed, and significant differences between groups were calculated by a Tukey test ($p < 0.05$). Different letters (a-d), capital letters between barley plants, and small letters between wheat plants above error bars represent groups based on significance.

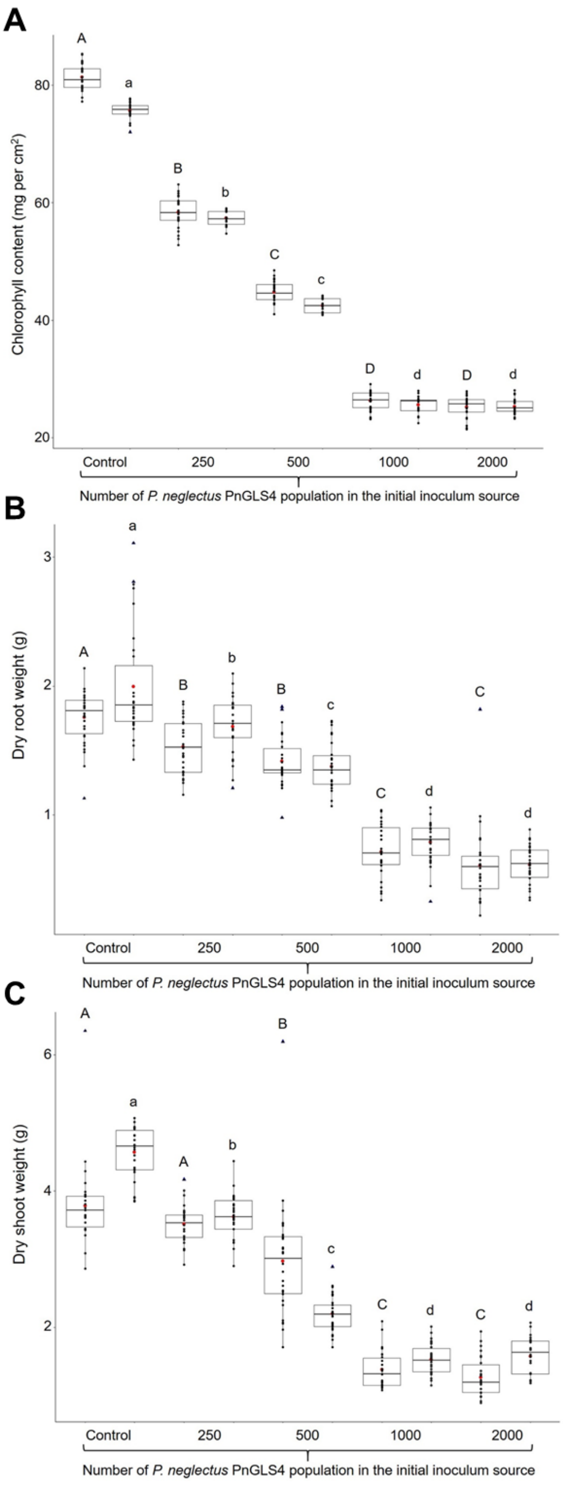

Supplementary Figure 8: Effect of varying *Pratylenchus neglectus* inoculum size on plant growth and physiological traits. Ten days after sowing, plants were inoculated with different numbers of *P. neglectus* isolate PnGLS4 (Pi: 250, 500, 1000, and 2000) individually and grown in the greenhouse for eight weeks after inoculation (Experiment 'B'). Roots were harvested eight weeks after inoculation. (A): Chlorophyll content, (B): Dry root weight, (C): Dry shoot weight. Individual and mean values are marked in black and red dots, respectively. The Upper and lower quartiles are separated by the median (horizontal line). Blue triangles represent outliers. Error bars represent the standard error of the mean from biological replicates. An ANOVA test ($p < 0.05$) was performed, and significant differences between groups were calculated by a Tukey test ($p < 0.05$). Different letters (a-d), capital letters between barley plants, and small letters between wheat plants above error bars represent groups based on significance.

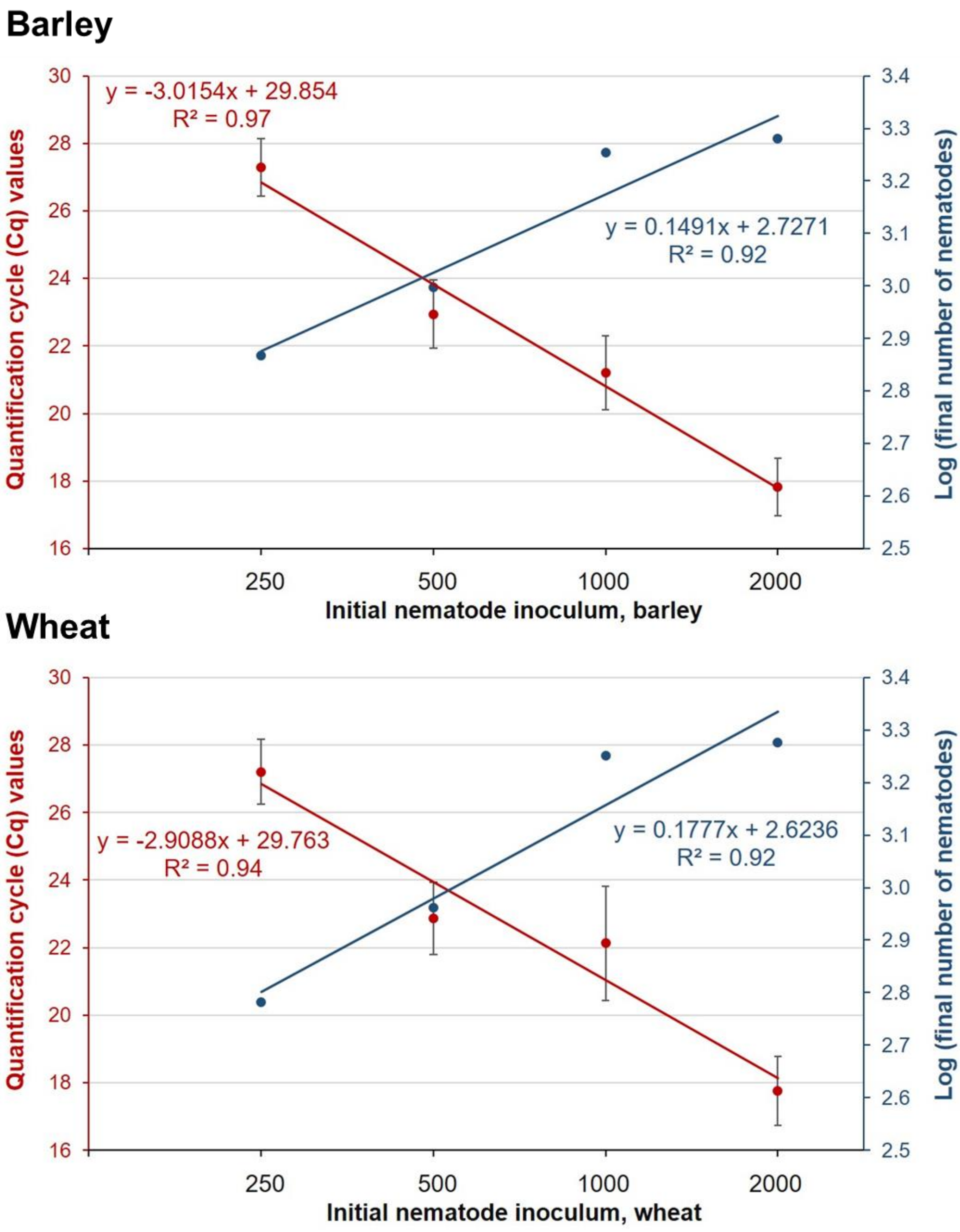

Supplementary Figure 9: Regressions between the initial inoculum, quantification cycle (Cq) values, and the logarithm transferred of the final number of nematodes. Plants were harvested eight weeks after inoculation with *P. neglectus*. Regressions were calculated with infection data from barley and wheat. The Neg1 primer combination was used. Each data point is the mean of fifteen biological repetitions of each treatment and three technical replication for each. Plants were inoculated with different numbers of *P. neglectus* per plant (250, 500, 1000, and 2000) when they were ten days old.

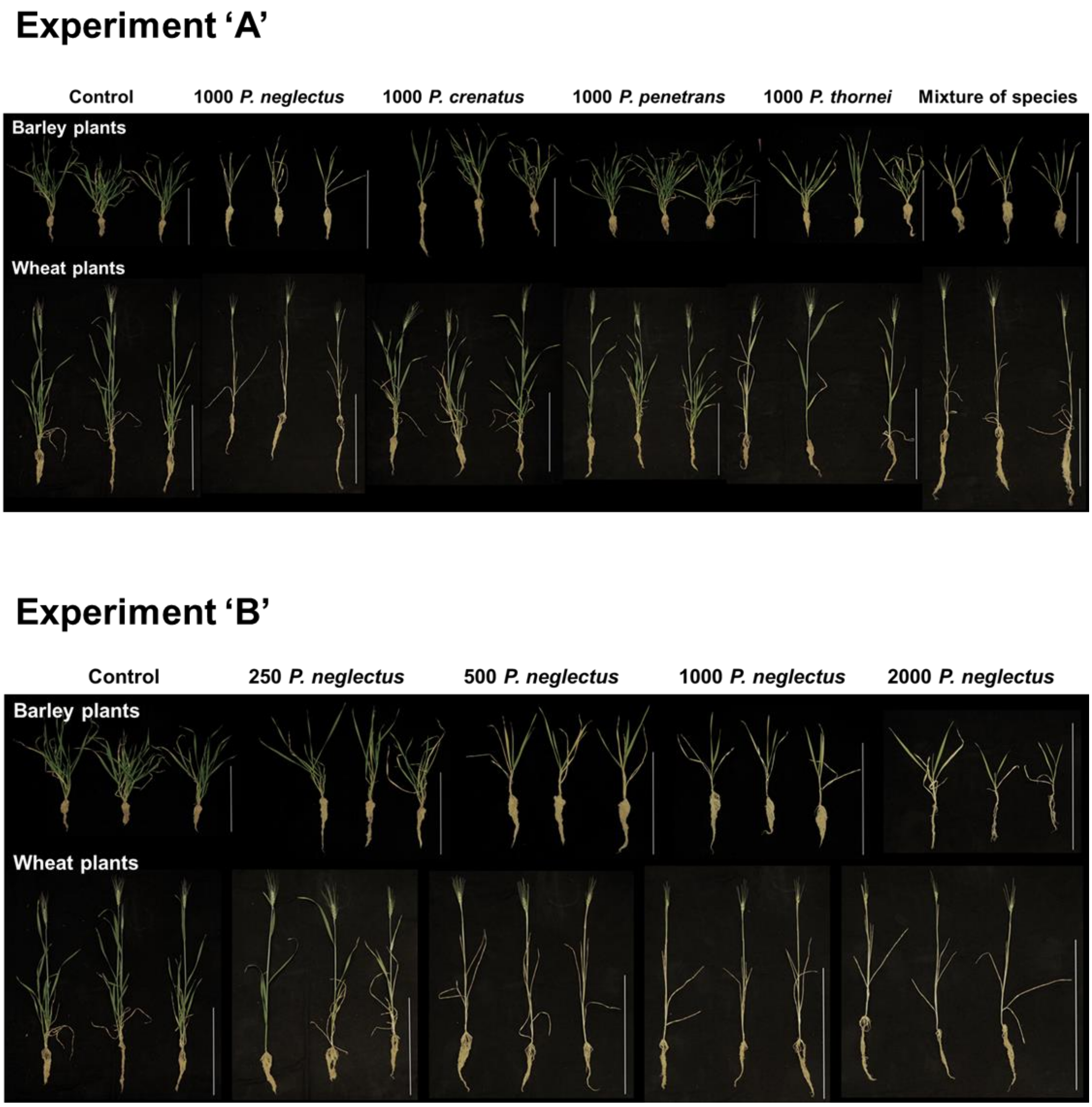

Supplementary Figure 10: Performance of plants in the greenhouse experiments 'A' and 'B'. Roots were harvested eight weeks after inoculation. Plants were infected with RLNs ten days after sowing and kept in the greenhouse under long-day conditions (16 hours of light) at 23°C during the day and 18°C at night. Scale bar: 30 cm.

Supplementary tables

Species	Acronym	Geographical Origin	Supplier
Pratylenchus neglectus	PnGLS4	Groß Lüsewitz, Germany	Julius Kühn-Institut (Münster, Germany)
Pratylenchus neglectus	PnItaly	Viterbo, Italy	Tuscia University (Viterbo, Italy)
Pratylenchus neglectus	PnH1	Haßloch, Germany	Julius Kühn-Institut (Braunschweig, Germany)
Pratylenchus neglectus	PnUK1	Mere, England	Julius Kühn-Institut (Münster, Germany)
Pratylenchus neglectus	PnL1	Lachapelle Ozerain, France	Julius Kühn-Institut (Münster, Germany)
Pratylenchus neglectus	PnL2	Lorup, Germany	Julius Kühn-Institut (Münster, Germany)
Pratylenchus neglectus	PnT1	Turkey	Julius Kühn-Institut (Münster, Germany)
Pratylenchus crenatus	PcH1	Heinrichshofen, Germany	Julius Kühn-Institut (Münster, Germany)
Pratylenchus crenatus	PcG1	Lorup, Germany	Julius Kühn-Institut (Münster, Germany)
Pratylenchus penetrans	PpW1	Witzenhausen, Germany	Kassel University (Kassel, Germany)
Pratylenchus penetrans	PpUK3	Mere, England	Julius Kühn-Institut (Münster, Germany)
Pratylenchus penetrans	PpI1	Lorup, Germany	Julius Kühn-Institut (Münster, Germany)
Pratylenchus thornei	PtS1	Slovenia	Julius Kühn-Institut (Münster, Germany)
Pratylenchus thornei	PtK1	Kulmbach, Germany	Julius Kühn-Institut (Münster, Germany)
Pratylenchus thornei	PtUK	Mere, England	Julius Kühn-Institut (Münster, Germany)
Meloidogyne incognita	Mi	Münster, Germany	Julius Kühn-Institut (Münster, Germany)
Meloidogyne hapla	Mh	Münster, Germany	Julius Kühn-Institut (Münster, Germany)
Heterodera schachtii	Hs	Kiel, Germany	Christian-Albrecht University (Kiel, Germany)

Supplementary Table 1: Plant parasitic nematodes used in this study.

Primer name (This study)	primer name (Published)	Sequence 5′ → 3′	Length (bp)	CG%	Reference	Amplicon (bp)	Annealing temperature (°C)	Sequence origin	Method
Neg1-fw	Pn-ITS-F2	GGCACTGTGCGAAGTGTCCG	20	65.0	(Yan et al. 2013)	234	61	ITS1 regions + 5.8S	RT-qPCR/ SYBR Green
Neg1-re	Pn-ITS-R2	TTAACACCTCAGGCGTCATGTAC	23	48.0					
Neg2-fw	F: β14Pnf1	TGACCACAACGCGCAGAACCAC	22	59.0	(Peetz and Zasada 2016)	293	65	β-1,4-endoglucanase	PCR
Neg2-re	R: β14Pnr2	GCCACGTCCACGTCCTGGGA	20	70.0					
Neg3-fw	PNEG-F1	CGCAATGAAAGTGAACAATGTC	22	40.9	(Yan et al. 2008)	144	60	28S rRNA D3 expansion	RT-qPCR/ SYBR Green
Neg3-re	D3B5	AGTTCACCATCTTTCGGGTC	20	50.0					
Neg4-fw	PNEG-F	ATGAAAGTGAACATGTCCTC	20	38.1	(Al-Banna et al. 2004)	290	63	26S rDNA D3 expansion region	PCR
Neg4-re	D3B-R	TCGGAAGGAACCAGCTACTA	20	50.0					

Supplementary Table 2: Primers used in this study. The sequences were derived from the literature and the National Center for Biotechnology Information (NCBI) database.

**Primer name**	**Cq value ± SD**				**Melting peak (°C)**
	P. neglectus DNA	*P. neglectus*-inoculated barley DNA	*P. neglectus*-inoculated wheat DNA	DNA of inoculated barley with a mixture of four species	
Neg1	17.74 ± 0.09	20.19 ± 0.05	21.40 ± 0.07	23.21 ± 0.09	81.50
Neg2	20.59 ± 0.11	21.98 ± 0.14	23.44 ± 0.12	22.35 ± 0.13	88.50
Neg3	17.75 ± 0.08	21.40 ± 0.07	22.55 ± 0.05	24.52 ± 0.17	90.50
Neg4	21.74 ± 0.21	25.62 ± 0.11	25.50 ± 0.18	25.30 ± 0.16	85 and 90.50

SD: Standard deviation

Supplementary Table 3: Quantification cycle (Cq) values and melting peaks from different *P. neglectus*-specific primer combinations. DNA concentration was measured from 1000 *P. neglectus* DNA (28.23 ng/µl), *P. neglectus*-inoculated barley DNA (858.7 ng/µl), *P. neglectus*-inoculated wheat DNA (932.5 ng/µl), DNA of inoculated barley with a mixture of four species (866.3 ng/µl) and using a Qubit.
